# Identification of Metal–Organic Frameworks for near Practical Energy Limit CO_2_ Capture from Wet Flue Gases: An Integrated Atomistic and Process Simulation Screening of Experimental MOFs

**DOI:** 10.1021/acscentsci.5c00777

**Published:** 2025-07-07

**Authors:** Ohmin Kwon, Marco Gibaldi, Kasturi Nagesh Pai, Arvind Rajendran, Tom K. Woo

**Affiliations:** † Department of Chemistry and Biomolecular Sciences, 6363 University of Ottawa, 10 Marie Curie Private, Ottawa, Ontario K1N 6N5, Canada; ‡ Department of Chemical and Materials Engineering, 3158 University of Alberta, 12th floor, Donadeo Innovation Centre for Engineering (ICE), 9211-116 Street, Edmonton, Alberta T6G1H9, Canada

## Abstract

Metal–organic framework (MOF) materials have attracted significant attention as solid sorbents for low energy CO_2_ capture with adsorption-based gas separation processes. In this work, an integrated screening workflow combining a series of atomistic and process simulations was applied to identify promising MOFs for a 4-step pressure-vacuum swing adsorption (P/VSA) process at three different CO_2_ flue gas compositions (6%, 15% and 35%). Starting from 55,818 unique experimentally characterized MOFs, ∼19k porous MOFs were investigated via atomistic grand canonical Monte Carlo (GCMC) simulations and machine learning model-based process optimizations to accelerate the screening of a large candidate database. Thousands of MOFs were identified for each of the CO_2_ compositions tested that could achieve within 4% of the practical energy limit of dry CO_2_ capture for the P/VSA process while still meeting the 95% CO_2_ purity and 90% recovery constraints. From this pool, 3D MOFs without open metal sites were subjected to the multicomponent (CO_2_/N_2_/H_2_O) GCMC simulations at 40% relative humidity. Based on these simulations, hundreds of MOFs were identified at each CO_2_ composition that could retain 90% of their CO_2_ capture at this humidity while also adsorbing a minimal amount of water. A geometric analysis of these high performing materials revealed that narrow, straight 1D-channels were a common structural motif for low energy wet flue gas CO_2_ capture with P/VSA.

## Graphical abstract

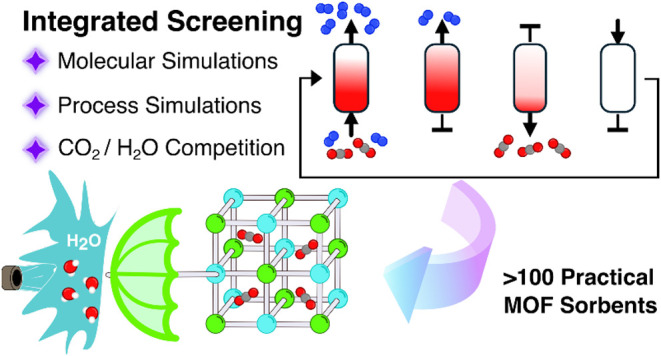

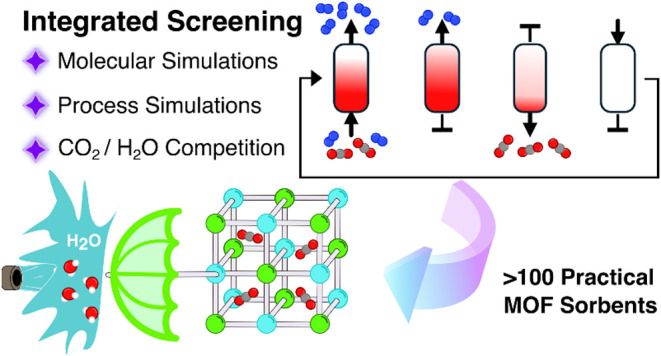

## Introduction

Nations worldwide have committed to the Paris Agreement which aims to reduce greenhouse gas emissions by 45% by 2030 and reach net zero emissions by 2050.^1,2^ All serious greenhouse gas mitigation strategies to reach the Paris targets include carbon capture and storage (CCS) as an integral part of the plan. The most mature technology for large scale CO_2_ capture from fossil fuel combustion employs an aqueous amine-based absorption process.^3–8^ Although the process has been deployed at full scale at many sites, this capture technology is very costly due to the high energy required to regenerate the sorbent and the cost to replace the amine sorbents which degrade over time.^4–9^ A leading alternative to liquid amine-based capture are adsorption technologies which use solid nanoporous sorbents such as pressure-vacuum swing adsorption (P/VSA) or similar processes.^10–12^ A key aspect in these systems is the selection of appropriate porous materials that can achieve low energy consumption within a given process configuration while maintaining the requisite CO_2_ purity and recovery targets (i.e., purity of the CO_2_ captured and fraction of CO_2_ recovered from the gas stream, respectively).^13^ In this respect, a class of materials called metal–organic frameworks (MOFs) have drawn significant attention because of their high diversity and potential tunability.^14–16^ Indeed over 100,000 MOFs, whose structures have been deposited in the Cambridge Structure Database (CSD),^17,18^ with an astonishing range of pore geometries and chemistries have been experimentally synthesized and characterized. The MOF CALF-20 has been commercialized for use in a proprietary steam swing process that is being scaled up for CO_2_ capture from cement making flues.^19^

To identify high performing MOFs for a given CO_2_ capture application, high-throughput computational screening workflows have become widely employed where atomistic grand canonical Monte Carlo (GCMC) simulations are used to compute a MOF’s gas adsorption properties.^20–25^ In such studies, MOFs are typically ranked in terms of their CO_2_ uptake capacity, CO_2_/N_2_ selectivity or similar equilibrium adsorption-based metrics where the actual process is usually not considered beyond specifying the adsorption and desorption conditions. However, recent studies have shown that these simple adsorption metrics do not correlate well to critical process level performance indicators,^13,26–28^ such as the energy consumption of CO_2_ capture, that are required to analyze the feasibility of the separation process. For example, A.K. Rajagopalan et al.^26^ performed case studies on several porous materials which revealed that Mg-MOF-74—previously considered a promising candidate for CO_2_ capture due to its high CO_2_ uptake capacity—actually exhibited higher energy consumption and lower productivity than UTSA-16 and Zeolite 13X, both of which have lower uptake capacities. Furthermore, without detailed process simulations one cannot even determine if a material will be able to meet the 95% purity and 90% recovery minimums that are often targeted for combustion flue gas CO_2_ capture. Therefore, detailed process simulations and optimization are essential in evaluating the true separation potential of adsorbents.

Adopting a screening workflow^29,30^ that integrates atomistic simulations with detailed process simulations, which take into account mass and heat transfer, Burns et al.^27^ examined the process performance of 1,632 experimentally characterized MOFs for CO_2_ capture from coal combustion flues (15% CO_2_) with a vacuum swing adsorption (VSA) process. The work demonstrated that 29 commonly used adsorbent metrics obtained from only atomistic simulations were not good predictors of the energy consumption of CO_2_ capture obtained from process simulations. Further, several MOFs akin to Mg-MOF-74—i.e. historically considered high performing due to their attractive CO_2_ uptake capacities—ranked outside of the top 150 MOFs in terms of process energy consumption. Interestingly, the MOF with the lowest energy consumption from this study, IISERP-MOF2,^31^ stood out because it had an unusually low N_2_ adsorption capacity and a notably lower energy consumption than any other MOF evaluated in the study.

One drawback of approaches incorporating process simulations is their long computation times, which typically prevent their use on large candidate databases. To address this issue, Pai et al.^32^ trained a rapid machine learning-accelerated process simulation model called MAPLE. They investigated the practical limits of CO_2_ capture from dry combustion flue gases of varying CO_2_ composition in a P/VSA process using MAPLE.^33^ Specifically, the authors examined the practical achievable performance limits of energy consumption and productivity in a 4-step P/VSA system (Figure 1) by varying the isotherm parameters of a hypothetical adsorbent while simultaneously optimizing the process conditions. It was found that solid sorbent-based P/VSA CO_2_ capture is not energetically competitive with liquid amine-based separations for combustion flues with CO_2_ concentrations below ∼10% even if the ideal adsorbent can be found. The study also compared the practical limits for a few real MOFs previously considered as best MOFs to the best hypothetical MOF; ultimately, showing that the best real MOF, IISERP-MOF2, has an energy of CO_2_ capture that is within 20% of the practical limit with an idealized adsorbent when the flue gas CO_2_ concentration is 10%. However, this ‘innovation gap’, as they termed it, drops to 2.5% when the CO_2_ concentration increases to 45%. In other words, for 45% CO_2_ flues, improving the adsorption properties compared to IISERP-MOF2 will only provide a 2.5% improvement in the energy consumption of the process. Thus, for high CO_2_ concentration gas stream, where P/VSA is likely to be the most energetically competitive, IISERP-MOF2, already exhibits near ideal adsorption properties for CO_2_ capture under dry conditions.

**Figure 1:**
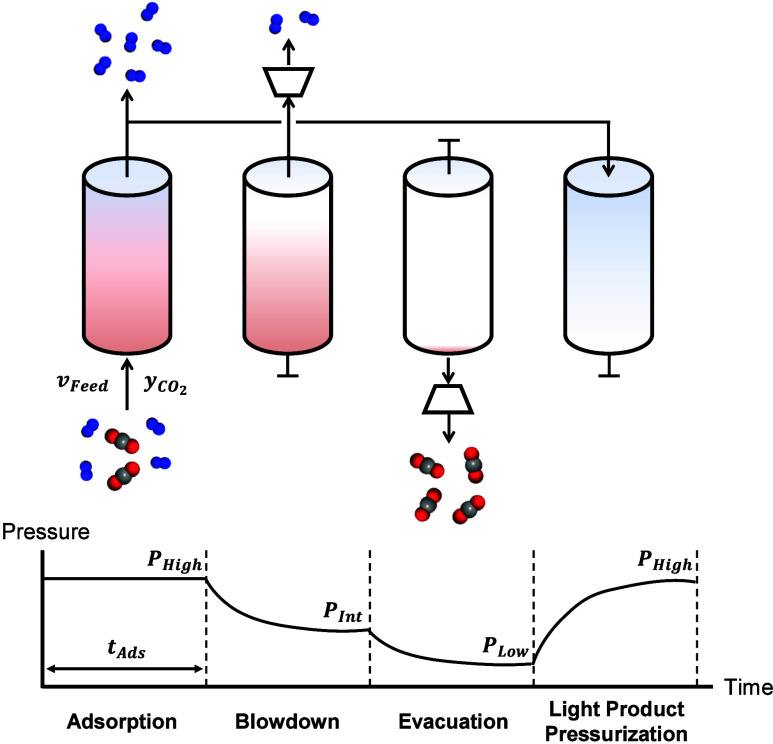
Schematic of the 4-step P/VSA process with light product pressurization (LPP) studied in this work. Red, gray, and blue spheres in the molecules represent oxygen, carbon, and nitrogen atoms, respectively.

However, important questions remain unanswered. First, whether IISERP-MOF2 is truly an exceptional material with near ideal adsorption properties for P/VSA or do many MOFs exist that can provide near ideal performance? Second, is simply considering the adsorption or process performance sufficient? As recent discussions of carbon capture^30^ have suggested, independently considering the atomistic or process performance is insufficient to assess the feasibility of a given material or process configuration in any practical sense. This is further exemplified by the increasing^34–40^ scrutiny of prospective CO_2_ sorbents’ hydrophobicity and general stability properties in contemporary experimental works. Many specifically emphasize characterizing how well the material retains its adsorption properties and crystallinity under harsh conditions such as high humidity, simulated process cycling, exposure to harsh chemicals and temperatures.^34–40^ From these reports, it is evident that any up-and-coming materials must maintain most of their adsorption capacity under even high humidity conditions that may appear intermittently in real process flue gas compositions. Consequently, to advance the conversation, effective computational screenings should regard as many stages of their implementation in the targeted process as possible; including but not limited to calculation of adsorption properties under realistic flue gas conditions via atomistic simulations, process optimization to evaluate process performance metrics, assessment of the sorbent’s chemical and mechanical stability under process cycling conditions, appraisal of the sorbent synthesizability and scale-up and many others.

To date, while several studies attempt to augment their screening efforts with one or two of these considerations, fewer works have attempted to consider the end-to-end practicality of their reported top-performing materials. For instance, numerous studies incorporate considerations of realistic flue gas conditions in their GCMC simulations,^41–45^ however, they do not proceed into any process modeling. These implementations have historically consisted of inclusion of high humidity ternary CO_2_/N_2_/H_2_O mixtures in the GCMC or prescreening of candidates based on metrics estimating their hydrophilicity (e.g., Henry’s Coefficient of H_2_O,^41,46^ H_2_O DFT interaction energies,^43^ presence of open metal sites,^42^ etc.) to eliminate unsuitable candidates. Conversely, other studies have factored in the process simulation components^47^ without gauging the impact of real, humid flues. Additionally, these prior computational studies all employed MOF databases beset by erroneous crystal structures as evidenced by recent studies discovering high incidence (nearing 50%) of chemically invalid substructures (i.e., overlapping particles, missing atoms, etc.) in popular databases.^48,49^ These findings produce considerable doubt in the accuracy of the simulated adsorption properties and process performance, ultimately hampering one’s ability to translate these screening results to the synthesis of real, performant sorbents. Beyond these drawbacks, almost all these studies decline to proceed toward the next step of practical realization involving screening of auxiliary performance factors (e.g., stability, synthesizability, etc.) that is demonstrated to be necessary in experimental studies. Recent accounts sought to automate the study of some of these factors through development of models predicting the thermal^50^ and/or water^51^ stability, as well as synthesizability,^52–54^ through data mining of literature data catalogues; however, these examinations have not yet been adopted by any existing multiscale screening studies to refine their top-performing sorbent recommendations.

Additionally, one would assume that many auxiliary properties will need to be proven before any computational candidate can be translated to promising real synthesis targets. Ultimately, the above-described state-of-the-art single property models cannot perform this derisking of top performing candidates alone; thus, barring futures development, literature survey by trained individuals remains the gold standard for shortlisting materials in wet flue gas CO_2_ capture processes. Thus, given this plethora of requirements, identifying a candidate is challenging and it is important to know whether the quantity of suitable MOFs with near ideal adsorption properties is large or extremely limited. For example, in the screening by Burn’s et al.^27^ there was a clustering of MOFs with energy consumptions ∼10% higher than that of IISERP-MOF2 but very few MOFs with energy consumptions lower than that.

With all these elements of a responsible screening protocol in mind, this work implements a truly comprehensive and integrated workflow implementing atomistic simulations, process optimization based on machine learning models, and a pragmatic analysis of the top performing candidates for their auxiliary materials properties. The proposed workflow was applied to efficiently screen over ∼56k experimental MOFs for CO_2_ capture from postcombustion flue gas with a 4-step P/VSA process with light product pressurization (LPP)^55,56^ as depicted in Figure 1. Importantly, these experimental MOFs originate from a database which excluded the frequently seen, chemically invalid crystal structures by way of chemically intuitive processing and error screening protocols;^57^ thereby significantly enhancing confidence that the simulated sorbents candidates match their true, experimental counterparts. Flue gases containing 6%, 15% and 35% CO_2_ under both dry and humid conditions (40% relative humidity, RH) were simulated and materials were ultimately evaluated based on the minimum energy achieved in the CO_2_ capture process, thereby providing a more realistic assessment of each material. Although it has been shown that P/VSA capture with CO_2_ concentrations less than ∼10% is not energetically competitive with liquid amine CO_2_ capture,^33^ we examine a 6% CO_2_ flue stream to ascertain if any informative trends in process performance exist. Further, for small-scale captures e.g. from mobile sources, P/VSA processes that are operated on electricity i.e., without the need for heat are preferred. One goal of this work is to answer the question as to whether experimentally characterized MOFs with near ideal adsorption properties for P/VSA CO_2_ capture are abundant or very rare. Additionally, if high performing MOFs are relatively abundant, then we aim to identify potential geometric and chemical features of the MOFs that give rise to low energy CO_2_ capture under humid conditions. An additional objective is to demonstrate how modern machine learning tools focusing on auxiliary sorbent considerations such as material stabilities can supplement the integrated screening to derisk any subsequently reported top-performers lists. Altogether, this integrated approach enables the identification of experimentally characterized, process-viable MOFs for CO_2_ capture under practical operating conditions.

## Methods

### Process Models

In this work we used a workflow that integrates atomistic GCMC simulations with process level simulations. We focus on pressure-vacuum swing adsorption (P/VSA) due to its lower energy demands and faster cycling compared to temperature swing adsorption (TSA). TSA may become competitive with efficient heat integration and future work could explore this avenue, however P/VSA currently offers a more energy-efficient and practical baseline for large-scale material screening. For each material, CO_2_ and N_2_ isotherms are computed and the process conditions are optimized to minimize the energy of CO_2_ capture while meeting the given purity-recovery targets (PRT).

One major limitation of detailed process simulations is the computational cost associated with solving the coupled partial differential equations until the system reaches a cyclic steady state for each material and process condition. Moreover, to identify the optimal process conditions that meet specific targets for each MOF, a process optimization procedure must be carried out, which requires evaluating process performance across thousands of different operating conditions for a single material. To circumvent this computational bottleneck, a machine learning-based surrogate model for process simulations was employed in this study. For comparison, a single simulation using detailed models typically requires about 5–10 min on a single core to compute the process performance for a single set of operating conditions. If ∼5,760 different process conditions are simulated, as is necessary in the proposed process optimization scheme, this would amount to approximately 480–960 core-hours for a single candidate. In contrast, the machine learning-based process optimizer requires only around 0.04–0.08 core-hours to complete an analogous task making it roughly 4 orders of magnitude faster. Thus, the ML-based process optimization framework enabled high-throughput screening of a large number of porous materials at the process level.

While the screening utilizes a machine-learning (ML) based process model, this ML-model^32^ is itself trained on a detailed process model outputs of 4-step P/VSA process with LPP developed by Haghpanah, R. et al.^58^ This process comprises (1) adsorption, (2) blowdown (3) evacuation, and (4) light product pressurization (LPP) steps as outlined in Figure 1. At the adsorption step, the feed flue gas is injected into the column to adsorb CO_2_ with a defined flow rate (*v*_Feed_) and adsorption pressure (*P*_HIGH_). Then, CO_2_ is mainly adsorbed in the column and most N_2_ breaks through the column, which forms the LPP stream. At the blowdown step, the primary goal is removal of residual N_2_ gas from the column to purify CO_2_ prior to the following step. Therefore, the inlet valve is closed and a vacuum pump draws gas from the outlet at a specified blowdown pressure (*P*_INT_). In the evacuation step, high-purity CO_2_ is obtained by another vacuum pump at a separate evacuation pressure (*P*_LOW_). As a final step to complete the cycle, the column is repressurized by the LPP stream from the initial adsorption step. The detailed process simulations consider heat, mass and momentum transfer equations, realistic vacuum pump efficiencies and flows related to aforementioned steps. The parameters defining the model such as the dimensions of the physical column, properties of the adsorbents and fluids, and decision variables for process optimization were tabulated in Supporting Information (Table S2). The key process performance indicators, such as CO_2_ purity, recovery, energy consumption and productivity, are evaluated once the simulation of the process has reached a cyclic steady state. These indicators are expressed as follows:

$$purity=\frac{\mathrm{moles of}\;{\mathrm{CO}}_{2}\;\mathrm{in the product}}{\mathrm{moles of}\;{\mathrm{CO}}_{2}\;\mathrm{and}\;N_{2}\;\mathrm{in the product}}\times 100\left[\%\right]$$

$$recovery=\frac{\mathrm{moles of}\;{\mathrm{CO}}_{2}\;\mathrm{in the product}}{\mathrm{moles of}\;{\mathrm{CO}}_{2}\;\mathrm{in the feed}}\times 100\left[\%\right]$$

$$energy\;consumption=\frac{\mathrm{energy consumption in the process}}{\mathrm{tonne of}\;{\mathrm{CO}}_{2}\;\mathrm{captured}}\left[\frac{\mathrm{kWh}}{\mathrm{tonne}}\right]$$

$$productivity=\frac{\mathrm{moles of}\;{\mathrm{CO}}_{2}\;\mathrm{in the product}}{\left(\mathrm{volume of adsorbent}\right)\times \left(\mathrm{cycle time}\right)}\left[\frac{\mathrm{moles}}{m^{3}\cdot s}\right]$$

A single-site Langmuir isotherm (SSL) model was applied to describe the competitive isotherm of CO_2_ and N_2_ during process modeling. It is worth noting that the isotherm of CO_2_ and N_2_ cannot be described by the SSL functional form for all the MOFs. Many recent works showed dual-site Langmuir (DSL) form, which contains additional parameters, better described the equilibrium loading on various materials. However, the SSL form can also fit the isotherms of most MOFs with a smaller number of parameters, which reduces the training cost significantly compared to the DSL form. Therefore, we used the SSL isotherm model to describe the competitive isotherm in this work considering both efficiency and accuracy.

Further, the detailed process model depends on a set of ideal assumptions, including: (1) The gas phase obeys the ideal gas law. (2) The flow in the column is described by an axially dispersed plug flow model. (3) The solid and gas phases are in thermal equilibrium in the column assumed to be adiabatic. (4) The adsorbent is assumed to be spherical particles with 2 mm diameter. (5) Mass transfer is described by a linear-driving force (LDF) model and it is assumed that the molecular diffusion in macropores determines the mass transfer. (6) The pressure drop in the column is described by Darcy’s law. In a more realistic case, there may be additional resistances due to film and micropores, which typically worsen the process performances. (7) No binder is assumed to avoid capacity loss and to evaluate all materials at the same pellet density, independent of crystal density. This levels the playing field and ensures comparisons are based purely on thermodynamic behavior. Therefore, the calculated results in this paper should be taken as the ideal results attainable when considering an ideal mass transfer efficiency. Importantly, the process model has been validated at the lab and pilot scale.^59^

This detailed process model was used to generate training data for a surrogate ML-process model, called MAPLE (Machine-assisted Adsorption Process Learning and Emulation) that has been described in a previous work.^32^ Therefore, its predictions reflect the assumptions and input space of the training data, which represent typical CO_2_ capture conditions and is validated to a high degree of accuracy as per our previous studies on adsorbent screening and lab scale testing of different adsorbents and process configurations. The neural network model was trained on 43,158 data points generated from the detailed model and was found to have a test set *R* ^2^ greater than 0.99 with respect to energy consumption. The range of the input features for the training set including isotherm parameters of sorbents and operating conditions is specified in Table S2. A more comprehensive discussion of the parameters and assumptions used in the detailed model and MAPLE are provided in the Supporting Information and previous analyses.^33,58^

In this work, minimizing energy consumption was selected as the target of process optimization based on following reason. An accurate estimation of productivity requires a more detailed understanding of adsorbent-specific diffusion kinetics and pellet morphology which are often uncertain or unavailable. While conservative and representative assumptions based on benchmark sorbents were utilized in the process model, productivity predictions inherently carry more uncertainty than energy prediction given the strong influence of intraparticle diffusion on productivity. In contrast, energy estimates are generally more robust to such assumptions, making them a more reliable basis for screening at this stage. Therefore, the minimum achievable energy consumption was chosen as criterion of process screening step in our screening workflow. A genetic algorithm (GA) combined with ML process models was utilized to determine the minimum achievable energy consumption meeting the defined purity and recovery constraints (95% and 90%). All machine learning (ML) models inherently exhibit some degree of error, and predictions in under-sampled regions of the training data may have higher uncertainty. While the overall input space may be well covered, insufficient sampling in certain subregions can lead the model to produce inaccurate predictions, such as unrealistically low energy consumption. The optimizer could then converge to this unphysical minimum, leading to misleading results. To prevent this, the energy was prohibited from going below threshold value for each flue gas composition. The threshold was set to be the practical energy limit determined from previous detailed process simulations^33^ at each flue gas composition. Further details regarding the process optimizations are available in the Supporting Information (Section S1.5).

### Database of MOFs

The majority of studies that computationally screen experimentally characterized MOFs utilize the CoRE MOF database^60,61^ that was first released in 2014 and significantly expanded in 2019. The latest CoRE database contains ∼19K experimental MOF structures taken from the CSD that were processed for atomistic simulations. Our recent analysis of the database reveals that ∼46% of its structures have severe structural errors such as missing (or duplicated) atoms, missing ligands or incorrect framework charge assignment.^49^ This was determined by a mixture of automated and manual comparison of the CoRE structures with each original publication which first characterized the structures. Screening such structures could lead to false materials ranking as the simulated properties on inaccurate, nonphysical structure definitions will not match the true materials’ performance in all likelihood. Further, the design space of the corresponding, correct structures remains largely unscreened as a result of the errors. To avoid this problem we sampled a subset of the recently developed MOSAEC database^57^ of experimental MOFs where a novel metal oxidation state-based algorithm was utilized to both prepare the structures for computation and to screen for the structural errors observed in the previous database. This database sampled from the CSD and subsequently processed (i.e., removal of solvent molecules, removal of disorder, identification of chemically invalid oxidation states, etc.) these experimental crystal structures to yield a database of high-fidelity—effectively 0% error rate—MOF structures for simulation. A subset of the MOSAEC database containing 55,818 neutral frameworks, including 1D and 2D coordination polymers, was considered in the following workflow. Care was taken to remove duplicate structures and related details can be found in the Supporting Information. As a next step, pore limiting diameters (PLDs) were calculated by Zeo++ (version 0.3.0)^62^ to filter nonporous materials. From the initial data set of ∼56K MOFs, structures with a pore limiting diameter (PLD) of less than 2.0 Å were removed. Although many screening studies use a much larger PLD cutoff closer to the kinetic diameter of CO_2_ (3.3 Å) to filter nonporous materials, we opted for a conservative value given that the MOF CALF-20, which is currently being commercialized for CO_2_ capture,^19^ has a relatively small PLD of 2.75 Å. This geometric filter resulted in a total of 23,599 MOFs for which GCMC simulations were performed. An overview of the screening workflow is given in Figure 2.

**Figure 2:**
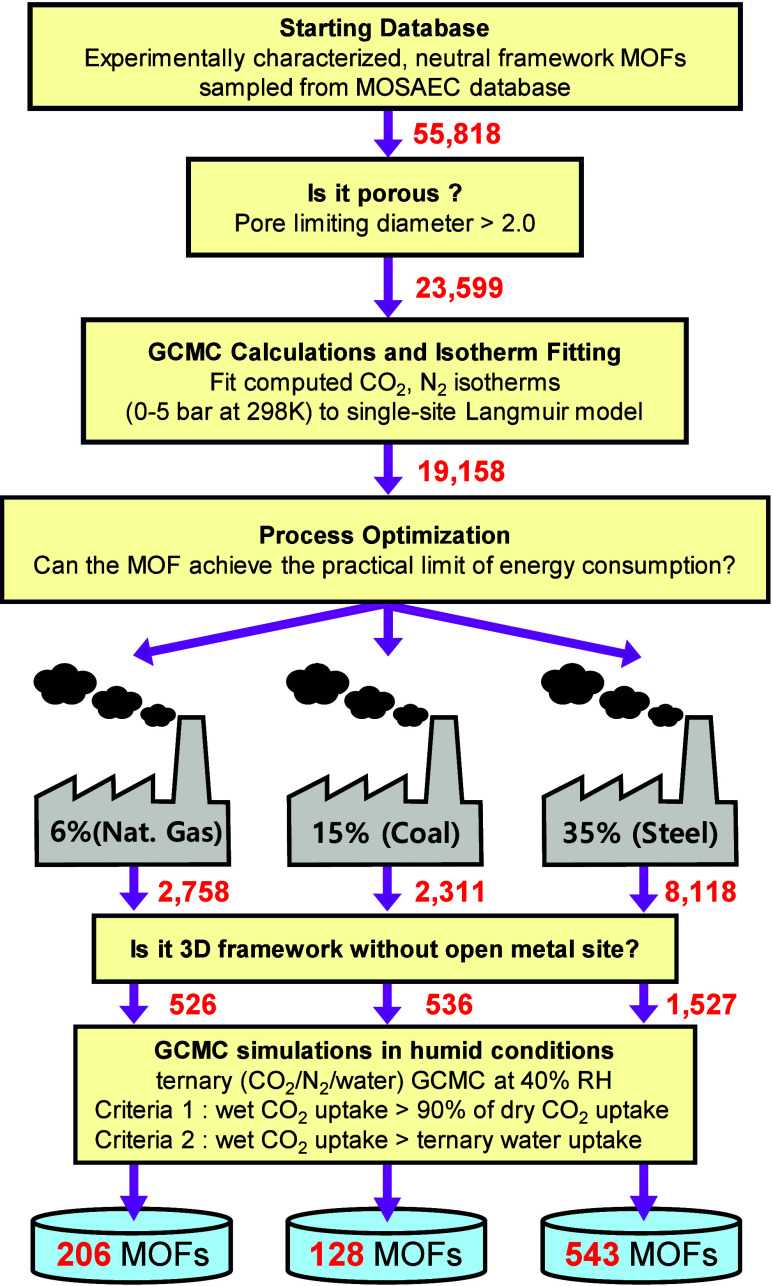
Overview of the multiscale screening procedure. The numbers represent the quantity of MOFs remaining at each stage of the screening workflow.

### Atomistic Simulations

Atomistic GCMC simulations were performed to obtain pure CO_2_ and N_2_ isotherms at 298 K and pressures up to 5 bar. Though it has been demonstrated that framework flexibility can impact simulated adsorption metrics,^63,64^ most MOFs observe a relatively minor impact on their adsorption properties. Given that performing full flexible calculations for each MOF is not feasible, a frozen framework assumption is applied to facilitate an integrated screening of this size. While recent studies^65–67^ have proposed machine learning models capable of predicting isotherms directly from MOF crystal structures, the predictive accuracy of these models remains limited by the diversity and quality of the training data, as well as the fidelity of the underlying GCMC simulations used for training. In contrast, GCMC simulations, though computationally more demanding, offer improved accuracy and remain relatively inexpensive compared to full process-level optimization simulations. Therefore, in this study, explicit GCMC simulations were employed to ensure reliability in the adsorption data.

The guest–host interactions were computed with atomic pairwise Lennard-Jones potentials and electrostatic interactions using partial atomic charges. For the single component isotherms of CO_2_ and N_2_, 10,000 cycles were used for both equilibration and production. For guest molecules, the parameters from Garcia-Sanchez, A. et al.^68^ and Provost, B et al.^69^ were used for CO_2_ and N_2_, respectively. UFF^70^ was used to describe the Lennard-Jones interaction parameters for all MOF framework atoms, while DFT-derived REPEAT charges^71^ were evaluated using the same protocol as described in previous work.^72^ GCMC simulations were first used to compute single component CO_2_ and N_2_ isotherms that were subsequently fit to a single site Langmuir model as given in eqs 1 and 2.

1 $$q_{i}=\frac{q_{sat}b_{i}C_{i}}{1+b_{i}C_{i}}=\frac{H_{i}C_{i}}{1+b_{i}C_{i}}$$

2 $$b_{i}=b_{0,i}e^{\left({-\Delta U}_{i}/RT\right)}$$

where *q*_*i*_, *b*_*i*_, *C*_*i*_, and Δ*U*_*i*_ represent uptake, equilibrium constant, concentration, and the internal energy, respectively, for the gas, *i* (CO_2_ or N_2_). The Henry constant *H*_*i*_ is defined by the product of *q*_sat_ and *b*_*i*_. The *q*_*i*_, *C*_*i*_, and Δ*U*_*i*_ values were taken or calculated from the GCMC results while *q*_sat_, *b*_0,CO2_, *b*_0,N2_ were obtained during the fitting process. The saturation uptake of N_2_ is assumed to be equal to that of CO_2_ to satisfy thermodynamic consistency.^73^ To ensure reliable results in the process optimization, fitting quality criteria were applied to exclude MOFs with poorly fitting isotherms. *R* ^2^ values (coefficient of determination) were calculated for both the CO_2_ and N_2_ isotherm fits, and only MOFs attaining *R*_CO_2__^2^ > 0.80 and *R*_N_2__^2^ > 0.80 were pursued further. Poor isotherm fits, resulting in their exclusion from screening, mostly occurred when the pores were too small to accommodate the gases, resulting in large fluctuations (noise) in the computed isotherm. Five isotherm parameters *q*_sat_, *b*_0,CO2_, *b*_0,N2_, Δ*U*_CO_2__ and Δ*U*_N_2__ were supplied to the process models.

For the last step of our workflow, multicomponent GCMC calculations for dry flue gas (CO_2_ and N_2_) and humid flue gas (CO_2_/N_2_/water) were conducted on promising MOFs with low energy consumptions from our process model to see if the MOFs possess CO_2_ retainability in the presence of water. For the dry flue gas, a total pressure 1 bar was used for all CO_2_ compositions (6, 15 and 35% of CO_2_). The same partial pressures of CO_2_ and N_2_ at each CO_2_ composition were used for humid flue gas with a fixed relative humidity (40%, 0.012628 bar). A four-site TIP-4P-Ew force field^74^ was applied to describe the interaction between the framework and water molecules. For the GCMC calculations involving water molecules, which typically require larger number of equilibration and production steps to thoroughly sample possible adsorbate configurations,^75,76^ 100 and 200 million steps were used for the equilibration and the production, respectively.

## Results and Discussion

Figure 2 shows an overview of the screening procedure used in this work, along with the number of MOFs remaining after each step. The screening was initiated from a collection of 55,818 neutral framework experimentally characterized MOFs sampled from the MOSAEC database.^57^ This includes structures in which only noncoordinating solvent molecules were removed and structures where both noncoordinating and coordinating solvent molecules were removed. Filtering small pore and nonporous MOFs (<2.0 Å) left 23,599 structures for which CO_2_ and N_2_ isotherms were computed via GCMC simulations. The isotherms were then fit to a single-site Langmuir adsorption model (eqs 1 and 2) resulting in 19,158 MOFs that were fed into the process optimizations with the MAPLE model. More than 80% of the MOFs which failed in fitting turned out to possess small pores yielding near zero gas uptake and/or noisy isotherms with similarly negligible uptakes. Further discussion relevant to these omitted, poorly fit isotherm can be found in Section S2 in the SI. Three different CO_2_ dry flue gas compositions were considered corresponding to natural gas combustion (6%), coal combustion (15%) and steel production (35%).

The results of the process optimizations are given in Figure 3, where the estimated Henry’s constants of CO_2_ and N_2_ of each MOF are plotted. The color of the data point represents its optimized energy consumption. It is important to note that the color map energy scale is different for each CO_2_ concentration. Data points in gray represent MOFs for which the 95% purity and 90% recovery targets (PRT) could not be met no matter what process parameters were applied. Based on the previous systematic analysis of the employed 4-step P/VSA process simulations under flue gas conditions in the range of CO_2_ composition (5–65%),^33^ its process performance bounds, namely in terms of the lowest achievable practical energy (PE), are well-known. As such, their MAPLE model analysis found the PE limit to be 544.9, 184.5, and 67.8 kWh/tonne in the case of 6, 15, and 35% CO_2_ flue gases. This integrated screening aims to optimize the sorbent materials space by finding those with process performance as close to this practical limit as possible which can then be subjected to progressively stricter filters. Hence, when optimizing the process for energy consumption minimization, we define the top performing materials as those achieving near practical limit energy consumption within the known prediction error margins of the employed surrogate ML model (±4%).^32^ The defined practical limit value at each composition determined from detailed process simulations^33^ served as the lower energy limit, as discussed in the Methods section. The darkest blue in the color maps for each CO_2_ concentration corresponds to candidates achieving energy consumptions at or near this PE limit.

**Figure 3:**
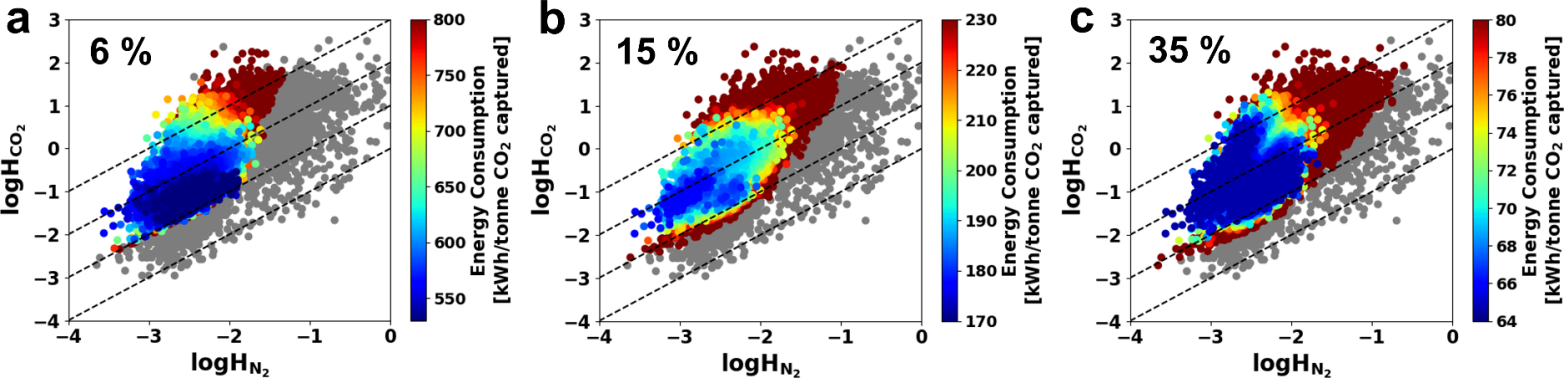
Plot of Henry’s law constant of CO_2_ and N_2_ for 19,158 experimental MOFs with optimized energy consumption in a P/VSA process for three different dry flue gas compositions: (a) CO_2_ 6% and N_2_ 94%, (b) CO_2_ 15% and N_2_ 85%, and (c) CO_2_ 35% and N_2_ 65%. The color mapping represents the minimum energy consumption achievable while satisfying the purity and recovery targets (PRT, 95% CO_2_ purity and 90% recovery). Note energy scale is different for each CO_2_ concentration. The gray points indicate that the MOF cannot meet purity-recovery constraints under any operating conditions. The dashed lines represent lines of constant selectivity calculated as the ratio of the Henry’s constants of CO_2_ and N_2_. Selectivity lines corresponding to values of 1, 10, 100, and 1000, respectively, are depicted from the bottom to the top of each figure.

The most striking feature of the plots shown in Figure 3 is the accumulation of dark blue colored data points describing the MOFs with moderate CO_2_ and N_2_ Henry’s constants that give near PE limits at each CO_2_ concentration. This suggests that for each CO_2_ concentration there are a considerable number of MOFs that can give low energy CO_2_ capture with this P/VSA process. More specifically, we find that 2,758, 2,311 and 8,118 MOFs were within 4% of the PE limit for the 6%, 15% and 35% CO_2_ cases, respectively. Previously we were only able to screen 1,632 MOFs due to the computational cost of the process optimization simulations, and only found 392 MOFs that could even meet the PRT.^27^ By using a much more efficient screening workflow that is accelerated with surrogate ML-models,^32^ a much larger pool of materials could be considered, allowing for the identification of thousands of MOFs that have adsorption properties that can give the practical limit of energy consumption under dry conditions. These results clearly address the first question raised in the introduction, demonstrating that IISERP-MOF2 is not unique in achieving near-ideal adsorption performance, and that many other MOFs can also deliver comparable performance.

The MOFs that are close to the PE limit are concentrated in a region of moderate Henry constants for both CO_2_ and N_2_ (Figure 3). When *H*_CO_2__ is too large, a sharp CO_2_ isotherm is observed at low pressure indicative of high CO_2_ interaction energy. This results in a lower desorption pressure being required, which leads to an energetically costly evacuation step. On the other hand, if *H*_CO_2__ is too small or *H*_N_2__ is too high, selective separation of CO_2_ from N_2_ becomes challenging, which prevents fulfillment of the 95% purity constraint. The gray points concentrated on the right side of Figure 3a-c clearly demonstrate the impact of N_2_ Henry’s constants on whether a MOF can meet the PRT, particularly at low CO_2_ concentrations. Also, the number of MOFs incapable of meeting PRT under any operating condition decreases as the CO_2_ composition increases (3,323–6%, 1,558–15% and 813–35%). In terms of selectivity, defined as ratio of the Henry’s constants of CO_2_ and N_2_, the MOFs approaching the PE limit are located in the region between values of 10 and 1000 across all three CO_2_ compositions. Further, the region corresponding to selectivity values below 10 solely comprises MOFs that either fail to meet the PRT or exhibit high energy consumption in the process despite meeting the PRT. Although our screening workflow is based on energy consumption, process optimization results for productivity maximization (Figure S3) also show similar trends and common region for the top performers with moderate Henry’s constant. These general trends are consistent with previous work using only hypothetical sorbents expressed in isotherm parameters without actual crystal structure. This work provides an actual mapping between the hypothetical isotherm space and the real MOFs.

To analyze the isotherm characteristics of the worst and best performing MOFs at each flue gas composition, the averaged volumetric isotherms of the MOFs which could not meet the PRT (gray line) and those achieving the PE limit (colored lines) are plotted in Figure 4 with the shaded regions spanning up to the first standard deviation. First, CO_2_ isotherms exhibiting sharp uptakes in the low-pressure region have a negative effect on PRT compliance in all cases and this impact increases as the CO_2_ concentration decreases. Moreover, N_2_ isotherms with high uptake do not assist in achieving PRT or lowering energy consumption. Rather, a moderate range of CO_2_ isotherms and relatively low N_2_ isotherms are required to achieve the practical energy limits. Interestingly, the dilute (6%) case tends to possess isotherms shapes closer to linear and narrower range of isotherms for both CO_2_ and N_2_ compared to the concentrated cases (15 and 35%). Above all, Figure 4 shows that a large range of isotherms can achieve energies close to the PE limit under dry conditions at all the CO_2_ compositions, further reinforcing that many MOFs, not just a few exceptional cases, possess adsorption characteristics capable of delivering near-ideal process performance.

**Figure 4:**
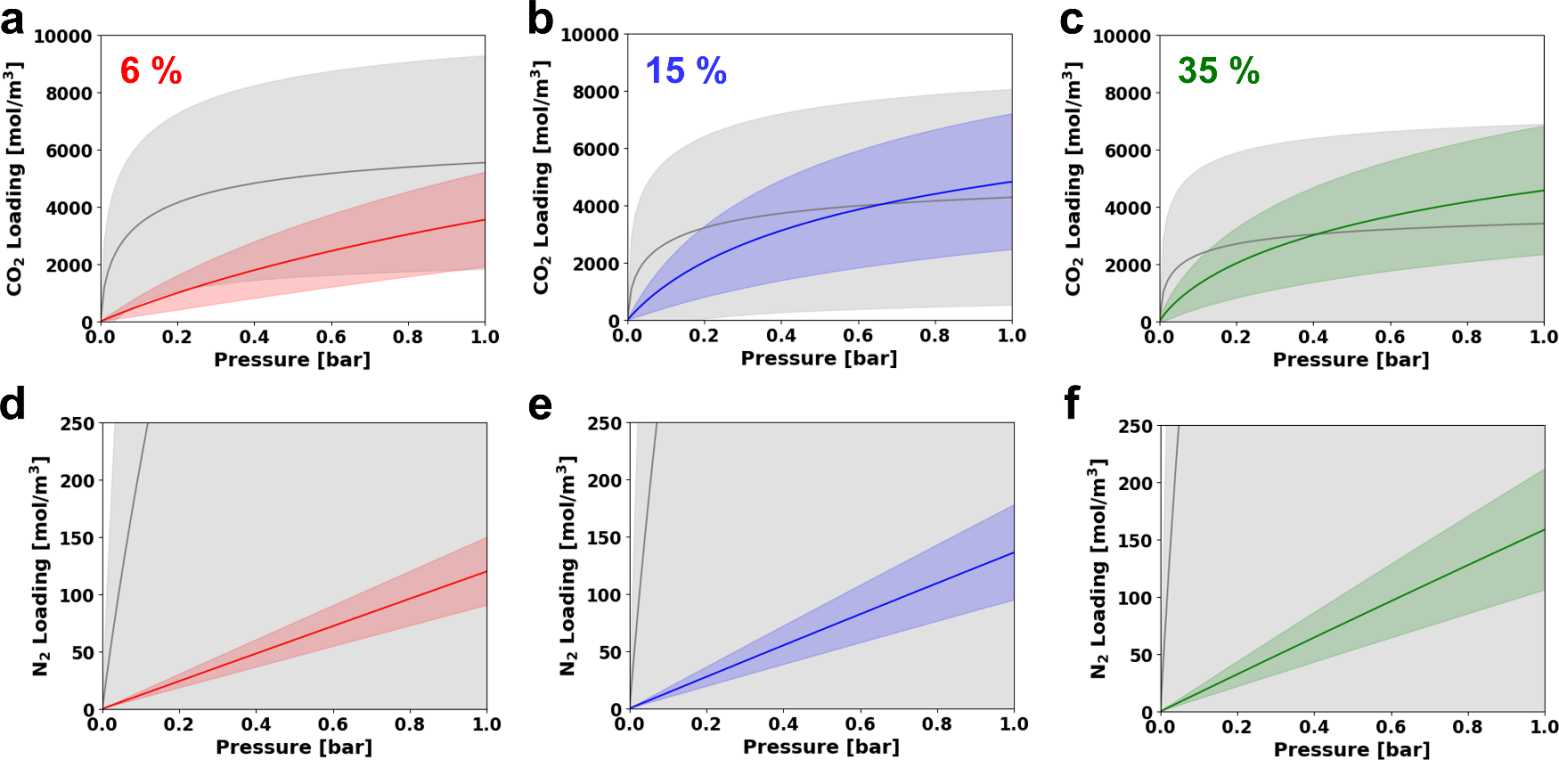
Averaged volumetric CO_2_ and N_2_ isotherms of the best performing MOFs and those that do not meet the purity-recovery targets (PRT) identified by process optimization. The first row displays CO_2_ isotherms at 6% (a), 15% (b), and 35% (c) CO_2_ flue gases, while the second row shows N_2_ isotherms at 6% (d), 15% (e), and 35% (f) CO_2_ flue gases, respectively. The colored isotherms signify the top-performers at each flue gas condition and the gray region represents isotherms of MOFs that could not meet the purity-recovery constraints under any operating conditions. The solid lines represent the averaged isotherm and the shaded regions span one standard deviation.

While many MOFs exhibit near-ideal performance under dry conditions in terms of process metrics, this alone is not sufficient for identifying truly viable materials for CO_2_ capture. A vital characteristic of a solid adsorbent for flue gas CO_2_ capture is that it is capable of functioning under humid conditions - otherwise energy must be expended to dry the gas stream before it is passed into the separation column. In many cases, even when the framework structure is stable toward water, a humid flue gas can degrade the CO_2_ capture performance by competing with CO_2_ for binding sites inside the pores. In this work, we have evaluated the impact of water on CO_2_ capture performance, by performing multicomponent (CO_2_, N_2_, H_2_O) GCMC simulations for each CO_2_ composition at 40% relative humidity (RH).

GCMC simulations of gas adsorption with water are very computationally demanding due to convergence issues^75–78^ and it is not unusual for such simulations to require 2 orders of magnitude more Monte Carlo steps than simulations without water.^76^ As such, starting from the MOFs that were computed to be within 4% of the PE limit at each CO_2_ concentration (2,758–6%, 2,311–15% and 8,118–35%), additional downselection was performed to reduce the number of MOFs which water simulations were performed on. First, MOFs with open metal sites were excluded, since these sites generally bind water molecules strongly giving rise to poor performance under humid conditions.^79^ Moreover, the classical potentials used in the GCMC simulations can be problematic when computing the guest interactions with open metal sites.^80,81^ We additionally excluded 1D and 2D coordination polymers since these are likely to have significant volume changes during the adsorption process. This may cause problems when structuring the materials (i.e., forming mm-sized beads) and these effects are not accounted for in the frozen framework GCMC simulations performed. This additional downselection gives a total of 526, 536 and 1,527 MOFs that were subjected to the multicomponent GCMC simulations at 6%, 15% and 35% CO_2_, respectively.

Results of the multicomponent GCMC simulations at 40% RH are given in Figure 5. The first row of Figure 5(a, b and c) plots the ratio of CO_2_ uptake at wet versus dry conditions as a function of the dry flue gas CO_2_ loading. MOFs whose CO_2_ uptake is significantly reduced in the presence of water will have a low ratio, while MOFs whose uptake is largely unaffected by water will have a ratio close to one or higher (green shaded region). To filter MOFs that are not likely to function well in humid conditions MOFs that have wet versus dry CO_2_ uptake ratios less than 0.9 (designated as the red shaded region in Figure 5(a, b and c) are excluded. The second row of plots in Figure 5(d, e and f), shows the ratio of wet flue gas CO_2_ uptake to H_2_O uptake as a function of the wet flue gas CO_2_ uptake. MOFs that have significant wet flue CO_2_ uptake, but whose CO_2_/H_2_O uptake ratio is less than 1 (red shaded area in Figure 5(d, e and f) will adsorb a substantial amount of water. As a result, we filter out these MOFs because a significant amount of energy will be required to remove water out of the column and separate water from CO_2_, thereby reducing the overall process efficiency.

**Figure 5:**
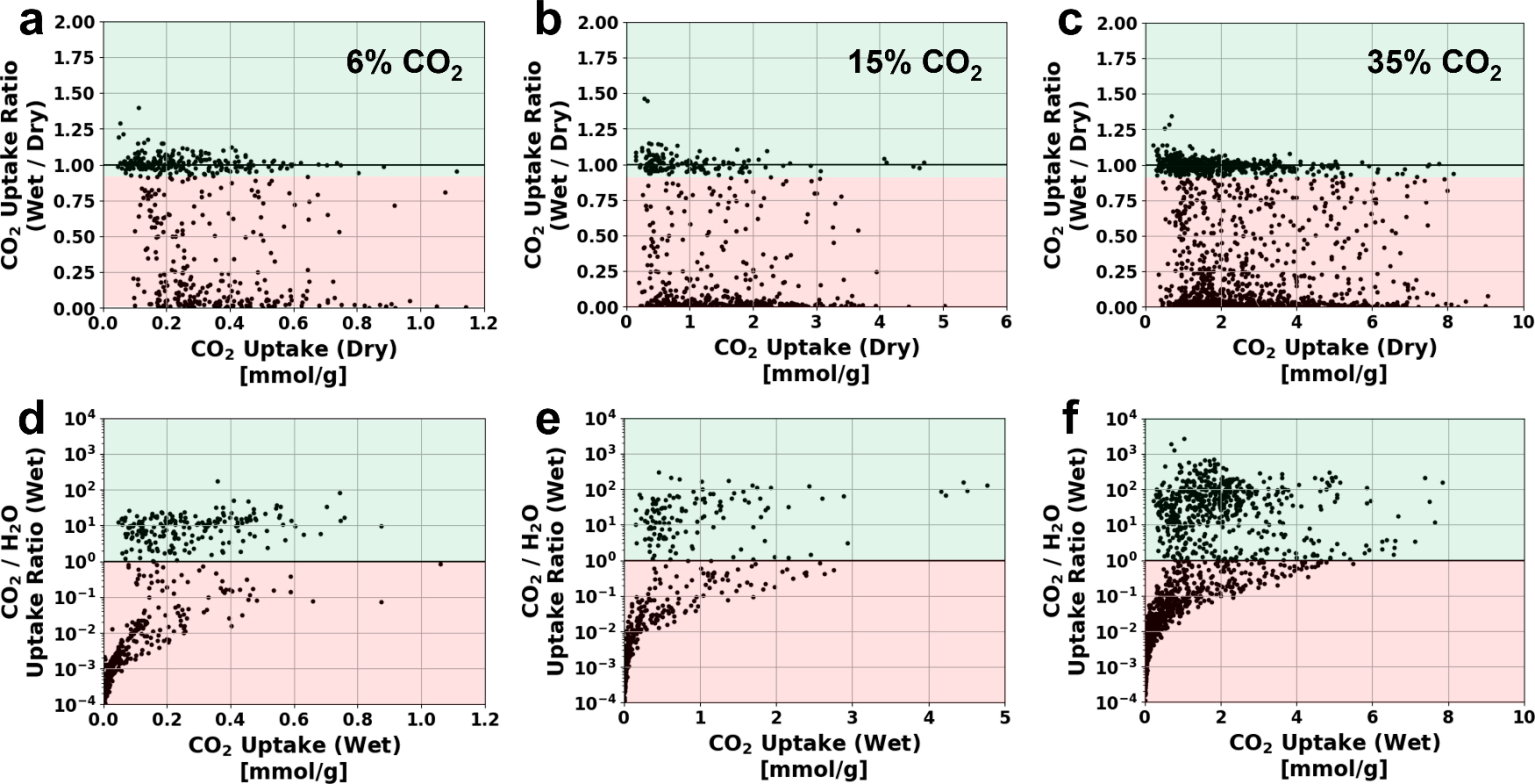
Water screening results for three different CO_2_ compositions based on three component GCMC simulations (CO_2_/N_2_/H_2_O) at 40% RH. The plots in the first row (a–c), give the ratio of wet (40% RH) and dry CO_2_ uptake, as a function of the dry CO_2_ uptake for (a) 526 MOFs at 6% CO_2_, (b) 536 MOFs at 15% CO_2_ and (c) 1,527 MOFs at 35% CO_2_. The second row of plots (d–f) give the ratio of wet CO_2_ molar uptake to water uptake as a function of the wet CO_2_ uptake at the three CO_2_ concentrations. The red shaded regions in all plots indicate the MOFs that were excluded from further consideration.

Down selecting MOFs based on the multicomponent gas adsorption simulations at 40% RH leaves 206 MOFs at 6% CO_2_, 128 MOFs at 15% CO_2_ and 543 MOFs at 35% CO_2_. A complete list of these structures is provided in the Supporting Information. These represent MOFs which have near ideal adsorption properties under dry conditions for the P/VSA process and whose CO_2_ adsorption under humid conditions does not change substantially. The fact that there are over a hundred MOFs identified for each CO_2_ concentration, is encouraging. We are, of course, aware that the outlined screening procedure employs many approximations and assumptions. For example in the atomistic simulations, there are approximations made in computing the interaction energies particularly with water, sampling issues such as assuming a frozen framework, and discrepancies between the perfect crystal which is simulated and the real materials containing defects and surface effects.^76^ It remains difficult to predict when accounting for such approximations (i.e., flexibility and/or adsorbate-induced structural changes, defects, etc.) will promote, demote, or ultimately not affect an individual candidate’s position in the overall ranking. Thus, rather than arbitrarily reducing the sorbent design space according to a property that is not proven to correlate with flexibility (or any other approximation), the proposed screening procedure elects to use the more rapid simulation approach and to be mindful of the potential pitfalls of this approximation when scrutinizing top performers feasibility in the final stages. Thus, we view these lists of MOFs as a starting point to look for potentially high performing MOFs for CO_2_ capture with the specified conditions and process.

One might expect the MOFs in these ‘final’ lists of candidate structures to share some common geometric or chemical features. Indeed, we found 62 MOFs that are common to all three CO_2_ concentrations. Beyond the duplicates in the three lists, we examined if there are any common geometric features in the identified MOFs by examining the top 50 MOFs ranked in terms of the CO_2_/H_2_O uptake ratio that also retain 90% of their dry CO_2_ uptake at 40% RH. For convenience, these lists of top-performing MOFs observed for each of the three studied CO_2_ concentrations will be herein referred to as the ‘top-50’. The only geometric feature that showed a bias in the top-50 for all three flue gas compositions was the pore limiting diameter (PLD). Figure 6a presents the distribution of pore limiting diameters (PLDs) for all MOFs that are within 4% of the PE limit for 6% CO_2_ dry flues, compared to the distribution of the top 50 MOFs. Figure 6b and 6c outline the same comparison for the 15% and 35% CO_2_ flues, respectively. When CO_2_ adsorption in the presence of water is considered, Figure 6 suggests that all three compositions prefer smaller PLDs, where the maximum in the PLD distribution shifts to smaller values. We note that Li et al.^82^ suggested that MOFs with smaller pores tend to discourage water molecules from clustering. However, the PLD is not a direct measure of the pore size, instead better corresponds to the ‘window’ size to the pore—though sometimes these may be similar in value. Another important pore characteristic, the largest cavity diameter (LCD), does not show any bias in their distributions (see Figure S4) as seen with the PLD. In general, a smaller window size is not likely to discourage water molecules from clustering and at this point it is unclear as to why this PLD bias exists for the high performing MOFs in the wet flue gas compared to those in the dry flue gas.

**Figure 6:**
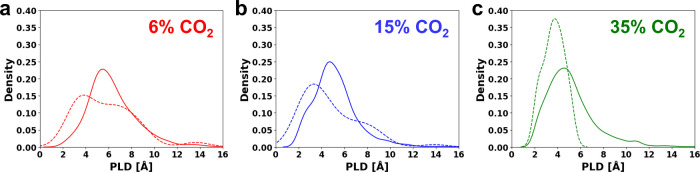
Distribution of pore limiting diameters (PLDs) for MOFs that are within 4% of the PE limit for CO_2_ dry flues (solid), compared to the distribution of the top 50 MOFs ranked in terms of the CO_2_/H_2_O uptake ratio (dashed) at 40%RH that also retain at least 90% of their dry CO_2_ uptake capacity. These distributions are shown for (a) 6% CO_2_, (b) 15% CO_2_, and (c) 35% CO_2_ flues.

The top 50 structures at each CO_2_ concentration were manually examined to identify any shared structural similarities and potential design principles for future P/VSA materials design. Of the 150 MOFs in total, only 117 were unique because some MOFs in the top 50 at one CO_2_ concentration were also found in the top 50 at another concentration. The common feature that stood out across these 117 MOFs was the presence of narrow, straight 1D channels. From these 117 unique MOFs, 48 MOFs (41%) could be characterized as having narrow, straight 1D channels with an average pore diameter of 4.8 Å. Examples are shown in Figures 7a, b and c. Interestingly, the MOFs CALF-20,^19^ NbOFFIVE-1-Ni,^83^ SIFSIX-18-Ni-β^84^ and IISERP-MOF2 ^31^ all have experimentally reported CO_2_ capture ability under humid conditions. All four of these MOFs possess narrow, straight 1D channels with pore diameters ranging from 2.6 to 3.7 Å. The structures for CALF-20, NbOFFIVE-1-Ni, and IISERP-MOF2 are shown in Figures 7d, e and f. The performances of these three experimental MOFs are summarized in Section S7. The fact that several MOFs that have been shown experimentally to retain their CO_2_ uptake under humid conditions share the same structural motif (small 1D channels) as a large fraction (41%) of MOFs identified in our screening lends some credibility to the screening protocol proposed in this work.

**Figure 7:**
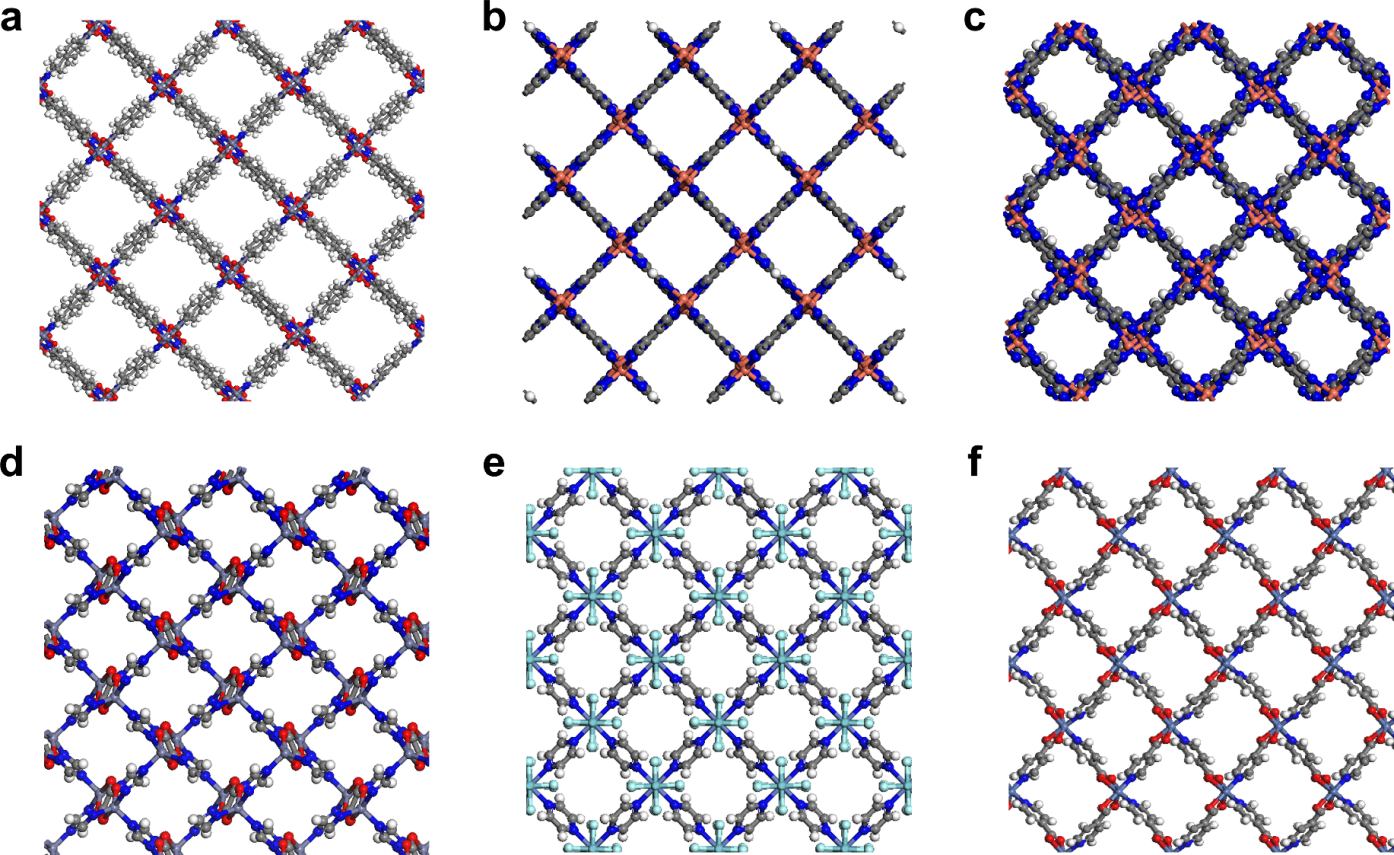
MOFs with 1D channels identified from the screening (a) MAF-X8 (FEHCIG), (b) MCIF-1 (JAWXEN), and (c) XAHROQ. MOFs that have been experimentally shown to retain their CO_2_ uptake capacity in the presence of water with 1D channels: (d) CALF-20, (e) NbOFFIVE-1-Ni, and (f) IISERP-MOF2.

An additional point of interest revolves around the interactions of each of these top-performers with water. MOFs have been defined as hydrophobic when their single component water isotherms are type V or VII.^85^ Type V isotherms are S-shaped, such that there is very low uptake at low RH, followed by a rapid uptake where water condenses in the pores. A type VII water isotherm is relatively flat and shows little water uptake at all RH ranges. We examined the original literature reporting the top 50 MOFs at each of the three CO_2_ compositions. Of the 117 unique MOFs, only three reported the material’s experimental water isotherms. However, all three cases reported type VII water isotherms indicative of highly hydrophobic materials.^86–88^ Given that most literature reporting MOF structures do not include water isotherms, our screening results and protocol provide opportunities to identify promising MOFs for humid flue gas CO_2_ capture in a manner that approximately coincides with the prior literature reports.

Another factor to be considered for practical realization is the stability of the sorbent materials. Many concepts contribute to the material stability assessment, such as activation stability, thermal stability and chemical stability when exposed to moisture or harsh chemical conditions. Though essential, no accurate and cost-effective computational methods for these stability predictions currently exist. Instead, one may either consult original experimental reports or employ state-of-the-art data-driven methods. While the former approach is accurate, experimental stability information is not consistently reported and further its retrieval is time-consuming. The latter methods train ML models on data collected through manual or automated text mining, thereby providing a cost-effective high-throughput option that can even function when no stability information exists for a given candidate. Thus, to further gauge the feasibility of the top 50 MOF lists reported, various categories of stabilities were predicted via recently reported ML classification models.^50,51^

Herein, emphasis is placed on water stability, yet the results of the other stability predictions are provided in the SI. This particular model outputs binary stability labels, but it also indicates a confidence level (i.e., confident versus uncertain) for each prediction. To first assess model accuracy, a small set (23 MOFs) possessing reported water isotherms was studied under the assumption that the material must be water stable to collect such data. Among this subset, 13 MOFs (56%) were classified with “confident” predictions—all of which were identified as water stable. Conversely, the remaining 10 (44%) observed “uncertain” predictions, with 7 ultimately being classified as water unstable. Thus, the “confident” predictions were deemed as a reasonable metric in the subsequent tests on the final 50 top-performing MOF lists. This analysis indicated that 78.9, 67.6 and 80% of MOFs with confident predictions were classified as water stable for 6, 15 and 35% CO_2_ composition cases, respectively. From the result, the majority of the final MOFs are suggested to possess water stability according to these model predictions. Regardless, one must be careful interpreting these predictions as the models can only access relatively small data sets compared to the total quantity of known MOFs. Since stability data is minimal and somewhat ambiguously defined, there is an inherent risk of overfitting and mislabeling. Therefore, these predictions should be regarded as first approximations and retrieving stability data from the original paper—or experimental characterization—is still recommended for final materials selection.

## Conclusions

Using an integrated atomistic and process simulation workflow, 23,599 experimentally characterized—and further, chemically accurate—MOFs were screened as solid sorbents for CO_2_ capture from combustion flues of different CO_2_ compositions deploying a 4-step P/VSA process. This approach is unique in its consideration of sorbent viability at all stages of screening, starting from the materials database fidelity to the atomistic and process performance calculations to the auxiliary materials properties such as stability. This screening was performed on dry flue gases with CO_2_ concentrations of 6%, 15% and 35% loosely corresponding to flues from natural gas combustion, coal combustion and steel production, respectively. For each MOF, the process parameters were optimized to deliver the lowest energy of CO_2_ capture while still meeting the 95% purity and 90% recovery constraints. Pai et al.^33^ have previously determined the lowest achievable practical energy limit of CO_2_ capture for the same P/VSA process. In that study, IISERP-MOF2 was identified to possess near ideal adsorption properties for the process since it is computed to be within ∼20% to 3% of this practical energy limit depending on the CO_2_ concentration. Part of the motivation of this work was to determine how unique IISERP-MOF2 is and to identify other possible candidate MOFs that also have near ideal adsorption properties for low energy CO_2_ capture within a P/VSA process.

In this screening, 2,758, 2,311 and 8,118 MOFs were identified as achieving process energy consumption on par with the practical energy limit for the 6%, 15% and 35% CO_2_ flues, respectively. Thus, thousands of MOFs at each of the three flue gas types could potentially yield low energy P/VSA CO_2_ capture similar to or better than IISERP-MOF2 if the flue gas is predried. We believe this is the first study to identify such a large number of experimental MOFs that can attain near practical energy limit performance for P/VSA. For example, in our previous screening work, only a total of 392 MOFs were found to meet the PRT under dry conditions irrespective of their energy consumption. Since drying the flue stream is energetically costly, the ideal MOF would retain its CO_2_ adsorption characteristics, while also adsorbing minimal water from a humid gas stream. To determine if any of the candidate MOFs could meet these additional criteria for wet flue gas CO_2_ capture, compute-intensive multicomponent GCMC simulations at 40% RH were performed. 206, 128, and 543 MOFs at 6%, 15% and 35% CO_2_, respectively were identified as potentially performing well under humid conditions. These were MOFs that retained their CO_2_ uptake capacity at 40% RH and that adsorbed a minimal amount of water. An examination of the ‘top 50’ of these MOFs at each CO_2_ concentration revealed that roughly 40% of the structures could be characterized as possessing narrow, straight, 1D-channels. This structural motif could therefore be targeted to further identify new MOFs as solid sorbents for low energy P/VSA CO_2_ capture from humid gas streams.

For a MOF to be used at industrial scale, it must not only have favorable adsorption properties under dry conditions, but it must also satisfy a number of stringent essential criteria. For example, it must function with wet flue gases, be extremely stable under operating conditions, its synthesis must be relatively low cost and scalable. Meeting all of these criteria is extremely difficult and currently CALF-20 is the only MOF we are aware of that is being commercialized for flue gas CO_2_ capture. In this work hundreds of MOFs have been identified that can potentially perform low energy CO_2_ capture under humid conditions with a 4-step P/VSA process. The abundance of potentially high-performing materials identified in this screening is encouraging for the ongoing search for a MOF that can meet all the additional requirements of a sorbent in practical applications.

## Supplementary Material





## Data Availability

Complete isotherm data from GCMC calculations and process optimization code written in MATLAB including machine learning surrogate process models are available at DOI: 10.6084/m9.figshare.29374544.

## Notes

Additional details of simulations, discussion of the isotherm fitting procedure, validation of process optimization using ML models, result of process optimization for productivity maximization, examples of 1D channel MOFs and performances for CALF-20, NbOFFIVE-1-Ni and IISERP-MOF2 (PDF)
Supplementary datasheet containing process results obtained at all flue gas compositions, including all fitted SSL isotherm parameters, optimized process conditions, predicted process performance indicators such as energy consumption, purity and recovery, and the lists of top performing MOF candidates (XLSX)

## §

R&D, Echeneidae Inc DBA Remora, 29834 Beck Road, Wixom, Michigan 48393, United States
