# Supplementary material for: Identification of Metal–Organic Frameworks for near Practical Energy Limit CO2 Capture from Wet Flue Gases: An Integrated Atomistic and Process Simulation Screening of Experimental MOFs
Source: ACS Cent Sci. 2025 Jul 7;11(8):1438–51. doi: 10.1021/acscentsci.5c00777 (PMC12395306; doi:10.1021/acscentsci.5c00777)
Supplement: Supplementary file 1 [file oc5c00777_si_001.pdf]

Supporting Information

Identification of Metal-Organic Frameworks for near Practical Energy Limit CO_2 Capture from Wet Flue Gases: An Integrated Atomistic and Process Simulation Screening of Experimental MOFs

Ohmin Kwon[1], Marco Gibaldi[1], Kasturi Nagesh Pai[2,3],

Arvind Rajendran[2,*] and Tom K. Woo[1,*]

[1] Department of Chemistry and Biomolecular Sciences,

University of Ottawa, 10 Marie Curie Private, Ottawa, Ontario, K1N 6N5, Canada

[2]Department of Chemical and Materials Engineering,

University of Alberta, 12th floor, Donadeo Innovation Centre for Engineering (ICE), 9211-116 Street, Edmonton, Alberta T6G1H9, Canada

[3]Current address: R&D, Echeneidae Inc DBA Remora,

29834 Beck Road, Wixom, 48393, Michigan, USA

*to whom correspondence should be addressed: twoo@uottawa.ca

Contents:

S1. Details of Multiscale Modeling

S1.1. MOF Database Preparation

S1.2. REPEAT Charge Calculation

S1.3. Grand Canonical Monte Carlo (GCMC) Simulation

S1.4. Isotherm Fitting

S1.5. Process Optimization

S1.6. Framework Dimensionality Calculation and Open Metal Site (OMS) Detection

S2. Additional Discussion of the Isotherm Fitting Procedure

S3. Validation of Process Optimization Using ML Models

S4. Result of Process Optimization Targeting Productivity Maximization at Three Different CO_2 Compositions

S5. The Distributions of the Largest Cavity Diameters (LCDs) for Both the Process Top Performers and the Final Top 50 MOFs across Each CO_2 Concentration Case

S6. Visualization of 1D Channel MOFs among the Final Top 50 MOFs at Three Different CO_2 Compositions

S7. Process Performances and GCMC Results under Humid Conditions for CALF-20, NbOFFIVE-1-Ni and IISERP-MOF2

References

S1. Details of Multiscale Modeling

S1.1. MOF Database Preparation

The majority of the MOFs studied in this integrated screening work were derived from the Cambridge Structural Database (CSD). Starting from more than 140k candidate MOF crystal structures, residual solvent molecules were removed to generate computation-ready structure using an in-house solvent removal procedure.[1] This procedure improves upon prior entries by including considerations of ligand formal charge and metal oxidation state when deciding whether molecules should be removed from the crystal structure. Chemical convention suggests that charged molecules are likely strong bound and typically crucial to the framework charge balance; thus, it is unrealistic to remove such species indiscriminately. Comprehensive details regarding the implementation and advantages of such an approach can be found in a separate work.[1] Following solvent removal, common structural errors—such as missing hydrogen atoms or counterions—were investigated by another in-house code (MOSAEC) which exploits metal oxidation state calculations to identify and remove MOFs which are likely to contain structural errors.[2] Next, pointwise distance distribution (PDD) analysis[3,4] implemented in average-minimum-distance python library was conducted to eliminate duplicate MOF structures (i.e. those possessing nearly identical crystal structures and chemical compositions). The threshold value applied to determine whether two structures are duplicates was carefully chosen through rigorous inspection of known duplicate crystal structure pairs and their corresponding PDD score. If two MOFs had a PDD value less than 0.15, they were regarded as duplicates and thus grouped together as equivalent entries. Once all the MOFs in a group with the same chemical formula were grouped, only a single MOF from each group was added to the database of screening candidates while the others were eliminated to avoid biasing later conclusions of the MOF structure-property relationship. Finally, pore limiting diameters (PLD) of those unique MOFs were calculated by Zeo++ (version 0.3.0) to eliminate nonporous MOFs which have PLD less than 2.0 Å.

S1.2. REPEAT Charge Calculation

Reliable charge calculations are necessary to correctly describe the adsorption behavior of CO_2, N_2 and H_2O, and produce high quality computational isotherms. Thus far, electrostatic potential (ESP) fitted charge methods are regarded as one of the most reliable methods of computing partial atomic charges. The REPEAT charge calculation[5] is one such ESP fitted charge methods that accurately reproduces DFT-calculated ESPs via a collection of atom-centered charges. The same procedure as described in previous work was used for the REPEAT charge calculations.[6]

First, DFT calculations on each MOF were conducted using VASP[7,8] with the PBE functional[9] and the projector augmented wave method[10] to generate the ESP. The energy cutoff for the plane-wave basis set of 300 eV was utilized and a threshold value of 10^{-5} eV was used as the convergence criterion of the electronic steps. The DFT-D3 method was used for the vdW dispersion energy correction term. For MOFs containing Gd, Dy, Ho, Er, Fe, Cr, Mn, Co and Ni, spin-polarized calculations (ISPIN = 2) were conducted while non-spin polarized calculations were conducted for any MOFs which did not contain those metal

atoms. Depending on unit cell sizes, two different grids for K point sampling were employed. A 3×3×3 k point grid was used for any unit cell containing a lattice vector less than 14 Å while only Γ-point was sampled in all other cases. Once the electrostatic potential was obtained from VASP calculations (LOCPOT file), REPEAT charge calculations were conducted using the Van der Waals scaling factor of 0.90.

S1.3. Grand Canonical Monte Carlo (GCMC) Simulation

Grand canonical Monte Carlo (GCMC) simulations at 298K were conducted using our in-house code–fastmc–to obtain gas uptakes. For reliable single component isotherm calculations, 11 pressure points ranging from 0.1 to 5.0 bar were selected for each guest molecule (see Table S1). 9 of these 11 pressures are concentrated on the low pressure region [0 1.2 bar] while the remaining two sampled high pressures to accurately obtain the saturation uptake. To describe the van der Waals interaction between the MOF frameworks and guest molecules, the Universal Force Field (UFF)[11] was applied for the frameworks while the guest molecule force fields defined by Garcia-Sanchez, A. et al.[12] and Provost, B et al.[13] were used for CO_2 and N_2, respectively. The Lorentz-Berthelot rule was used to describe interactions between different atom types. To calculate electrostatic interactions, REPEAT charges were used with Ewald summation. Within the GCMC simulations, 10,000 cycles were used in both the equilibration and production stages. 12.5 Å was used for a Lennard-Johns cutoff radius and Peng-Robinson equation of state was used to calculate fugacity.

Table S1. 11 Pressure Points for GCMC Calculations

Index	1	2	3	4	5	6	7	8	9	10	11
P [bar]	0.01	0.1	0.2	0.3	0.4	0.5	0.7	0.9	1.1	3.0	5.0

To determine if the promising MOFs in process performance have CO_2 retainability under humid conditions, multi-component calculations were performed for both binary (CO_2 and N_2) and ternary mixtures (CO_2/N_2/H_2O). For the binary mixture (dry flue gas), partial pressures of 0.06/0.94 bar, 0.15/0.85 bar and 0.35/0.65 bar (CO_2/N_2) were used for flue gases with CO_2 compositions of 6, 15 and 35%, respectively. The same partial pressures of CO_2 and N_2 at each CO_2 composition were used for the ternary mixture (humid flue gas) with a fixed relative humidity (40%, 0.012628 bar). The TIP-4P-Ew forcefield[14] was used for describing water–MOF interactions. For GCMC calculations involving water molecules which typically require a larger number of equilibration and production steps to properly sample different adsorbate configurations, 100 and 200 million steps were selected for the GCMC equilibration and production stages, respectively, to obtain more reliable results.

S1.4. Isotherm Fitting

To calculate the process performance using process calculation, a competitive isotherm (Eq (1)) must be calculated from pure component isotherm parameters. Once each pure component isotherm for CO_2 and N_2 was obtained from GCMC calculations, the isotherm

parameters were fitted to a single site Langmuir model. (SSL, Eq 2 and 3)

$$q_i^* = \frac{q_{sat}\, b_i C_i}{1+ b_{CO_2} C_{CO_2} + b_{N_2} C_{CO_2}} \quad (1)$$

$$q_{SSL,i}^* = \frac{q_{sat}\, b_i C_i}{1+ b_i C_i} \qquad i = CO_2, N_2 \quad (2)$$

$$b_i = b_{0,i}\, e^{\frac{-\Delta U_i}{RT}} \quad (3)$$

$$\Delta U_i = \Delta H_{ads,i} + RT \quad (4)$$

In these equations, q_i^* is the equilibrium loading of component i in mixture gas of CO_2 and N_2, $q_{SSL,i}^*$ is the equilibrium loading of pure component i. Using equation 2 and 3, and GCMC results, the isotherm parameters q_{sat} and $b_{0,i}$ were obtained. The average values of the heat of adsorption from GCMC calculations for different pressure in the same MOF were used as $\Delta H_{ads,i}$ values and the enthalpy values were converted to the internal energy change (ΔU_i) using Equation 4. To get reliable isotherm parameters, adjusted R^2 values were imposed as fitting criteria. A threshold value of 0.80 was selected for both CO_2 and N_2 isotherm fitting. Due to this fitting criteria, computational isotherms which are too noisy were excluded from successive analyses. The obtained five isotherm parameters of pure component isotherm (q_{sat}, b_{0,CO_2}, b_{0,N_2}, ΔU_{CO_2}, ΔU_{N_2}) from GCMC results were subsequently used as inputs for the process model calculations.

S1.5. Process Optimization

To evaluate the performance of each material at the process level, the minimum achievable energy consumption was calculated for each MOF using a process optimizer wherein a genetic algorithm is combined with a process model. A pressure-vacuum swing adsorptive process (P/VSA) with light product pressurization (LPP) process is considered in this work. The parameters utilized in the model include dimensions of the physical column, the properties of adsorbents and fluids, and decision variables for process optimization which are summarized in Table S2. Other details including model equations, boundary conditions and the key process performance indicators (KPIs) are calculated can be found in the previous papers.[15] A detailed process model requires solving coupled partial differential equations (PDEs) with nonlinear algebraic equations including mass and energy balance equations until the cyclic steady state is reached. However, the process optimization using the conventional detailed model is computationally expensive. For example, it takes ~960 core*hr per MOF to optimize the operating conditions when we consider 30 generations and 192 individuals for a generation and assume each process calculation takes approximately 10 minutes. Therefore, this process optimization typically represents a bottleneck in this workflow. To avoid this time-consuming task, a machine-assisted adsorption process learning and emulation (MAPLE) framework[16] written in MATLAB was engaged to perform the process optimization. In other words, the time-consuming detailed model was replaced by faster surrogate ML models to predict four P/VSA performance indicators (i.e. purity, recovery, energy consumption and productivity).

These machine learning (ML) models were trained on the results of 43,158 process simulations generated by the corresponding detailed model.[17] Comprehensive information concerning this ML model is analogous to what is reported in previous papers[15] except the fact that we extended the training set range of Q_{sat} value from [0 10] to [0 28.3] to cover more isotherm space. The MAPLE model architecture consists of a multilayered neural network model trained using a Bayesian regularization technique. It comprises 3 hidden layers with 30 nodes in each layer, and each performance indicator was trained using an individual neural network. The training data was split into 90/10 training and validation split. The training and validation MSE were monitored during training to avoid overfitting and an early stopping criterion was additionally implemented if the validation MSE showed an consistently increasing trend. Otherwise, the training was stopped after 200 epochs. The trained model was then tested against an independently generated test set comprising of 1000 samples and the R^2 value for this step was greater than 0.99 for each of the performance indicators.

Compared to the original paper of the process optimization using MAPLE model, the initialization step of the GA optimizer for the process optimization was modified to obtain stable results. Originally, only Latin hypercube sampling was conducted; however, we found that, without modification, one would need to perform multiple replicates of the process optimization to determine the true minimum achievable energy. In this work, a grid sampling technique was conducted along all the design variables by taking advantage of the rapid ML model inference. For example, there are 5 design variables, $(t_{Ads}, P_H, P_{INT}, P_L, v_{Feed})$ and each of their ranges was equally divided into 20 intervals to yield 20^5 grid points for the design variables which were subsequently fed into the ML models to predict the purity, recovery, energy and productivity KPIs. The total evaluation time of MAPLE model for all such grid points only required roughly 20 seconds per MOF candidate. From these results, the 96 best-performing design variable grid points were carried forward to form half of the initial population in the GA operating condition optimization, while the remaining half was randomly sampled according to the previous Latin hypercube sampling approach. Ultimately, the GA optimizer uses 192 individuals in a generation and goes up to 30 generations to determine the optimized operating conditions toward the minimum achievable energy. The inclusion of this seeding from grid point sampling yields a more stable process optimization result than previous efforts, thereby allowing a single run to be sufficient to find the minimum achievable energy consumption.

The following fitness functions (or penalty functions) were considered by the GA process optimizer when searching for a minimum achievable energy consumption meeting purity (95%) and recovery (90%) requirements:

If $Pu \le 0$ or $Re \le 0$ or $Pu \ge 102$ or $Re \ge 102$ or $En \le practical\ limit\ threshold$ or $Pr \le 0$,

$$F(En, Pu, Re) = 6000$$

Else,

$$F(En, Pu, Re) = \frac{En}{100} + 2000(Max(0, 0.95 - \frac{Pu}{100}))^2 + 2000(Max(0, 0.90 - \frac{Re}{100}))^2$$

Pu, Re, En and Pr means purity, recovery, energy and productivity calculated from ML models.
"practical limit threshold" for 6, 15 and 35% : 523.5, 176.6 and 64.6 kWhe/tonne, respectively.

Table S2. Parameters Used for Detailed Model Simulations

Parameter	Type*	Value
**Column properties**		
bed length, L [m]	Fixed	1.0
Column inner radius, r_{in} [m]	Fixed	0.14
Column outer radius, r_{out} [m]	Fixed	0.16
Particle voidage, ε_p [-]	Fixed	0.35
Particle radius, r_p[m]	Fixed	1×10^{-3}
Density of the column wall, ρ_w [kg m^{-3}]	Fixed	7800.00
Bed voidage, ϵ [-]	Fixed	0.37
Tortuosity factor, τ [-]	Fixed	3.00
**Fluid properties**		
Effective heat conduction coefficient, K_z [J m^{-1} s^{-1} K^{-1}]	Fixed	9.03×10^{-2}
Thermal conductivity of the wall, K_w [W m^{-1} K^{-1}]	Fixed	16.00
Inside heat transfer coefficient, h_{in}	Fixed	0.00
outside heat transfer coefficient, h_{out}	Fixed	0.00
Gas specific heat capacity, $C_{p,g}$ [J kg^{-1} K^{-1}]	Fixed	1010.60
Adsorbed-phase specific heat capacity, $C_{p,a}$ [J kg^{-1} K^{-1}]	Fixed	1010.60
Adiabatic constant, γ [-]	Fixed	1.40
Universal gas constant, R_g [m^3 Pa mol^{-1} K^{-1}]	Fixed	8.314
Fluid viscosity, μ [kg m^{-1} s^{-1}]	Fixed	1.72×10^{-5}
Molecular diffusivity, D_M [m^2 s^{-1}]	Fixed	1.60×10^{-5}
**Adsorbent properties**		
Adsorbent specific heat capacity, $C_{p,s}$ [J kg^{-1} K^{-1}]	Fixed	1070.00
Density of the solid particle, ρ_w [kg m^{-3}]	Variable	800.00 to 1200.00
Adsorption saturation capacity, q_{sat} [mol kg^{-1}]	Variable	0.50 to 28.5
Adsorption equilibrium constant of CO_2, b_{0,CO_2} [m^3 mol^{-1}]	Variable	10^{-14} to 10^{0}
Adsorption equilibrium constant of N_2, b_{0,N_2} [m^3 mol^{-1}]	Variable	10^{-12} to 10^{0}
Internal energy of adsorption of CO_2, ΔU_{CO_2} [kJ mol^{-1}]	Variable	–7.00 to –48.00
Internal energy of adsorption of N_2, ΔU_{N_2} [kJ mol^{-1}]	Variable	–3.00 to –25.00
**Process properties**		
Feed temperature, T_F [K]	Fixed	298.15
Blowdown step vacuum pump flow rate, υ_{BLO} [m s^{-1}]	Fixed	0.59
evacuation step vacuum pump flow rate, υ_{EVAC} [m s^{-1}]	Fixed	0.90
Pressurization step exponential pressure history term, α_{PRESS} [s]	Fixed	0.5
Vacuum pump efficiency, η [%]	Variable	$\frac{15.84\text{P[bar]}}{1+19.8\text{P[bar]}}$
Adsorption step time, t_{ADS} [s]	Variable	10.00 to 110.00
High pressure, P_{HIGH} [bar]	Variable	1.00 to 5.00
Intermediate pressure, P_{INT} [bar]	Variable	0.07 to 4.00
evacuation pressure, P_{LOW} [bar]	Variable	0.01 to 1.00
Feed rate, v_{FEED} [m s^{-1}]	Variable	0.10 to 1.50
CO_2 feed composition, y_{CO_2} [-]	Variable	0.05 to 0.65

* those indicated as a "Variable" are employed when training the MAPLE framework and used as decision variables

S1.6. Framework Dimensionality Calculation and Open Metal Site (OMS) Detection

To consider more realistic and practical MOFs during the integrated screening, 1D and 2D coordination polymers were excluded using a script which calculates the dimensionality of the MOF framework. This algorithm[18] analyzes how the smallest repeating unit of a given crystal structure evolves upon various degrees of polymer expansion across three dimensions. A comparison of the lengths of the minimum bounding box needed to fully contain the two differently expanded polymeric units determines in which dimensions the framework grew upon expansion, thereby allowing for identification of the framework's dimensionality. A python library called omsdetector[19] was used to determine whether a given MOF structures possesses open metal sites. This tool analyzes the coordination geometry (structural parameter, τ) at each metal atom site to identify vacant bonding positions according to each atom's expected bonding conventions. Only 3D MOFs without OMS were subjected to the final water screening simulations.

S2. Additional Discussion of the Isotherm Fitting Procedure

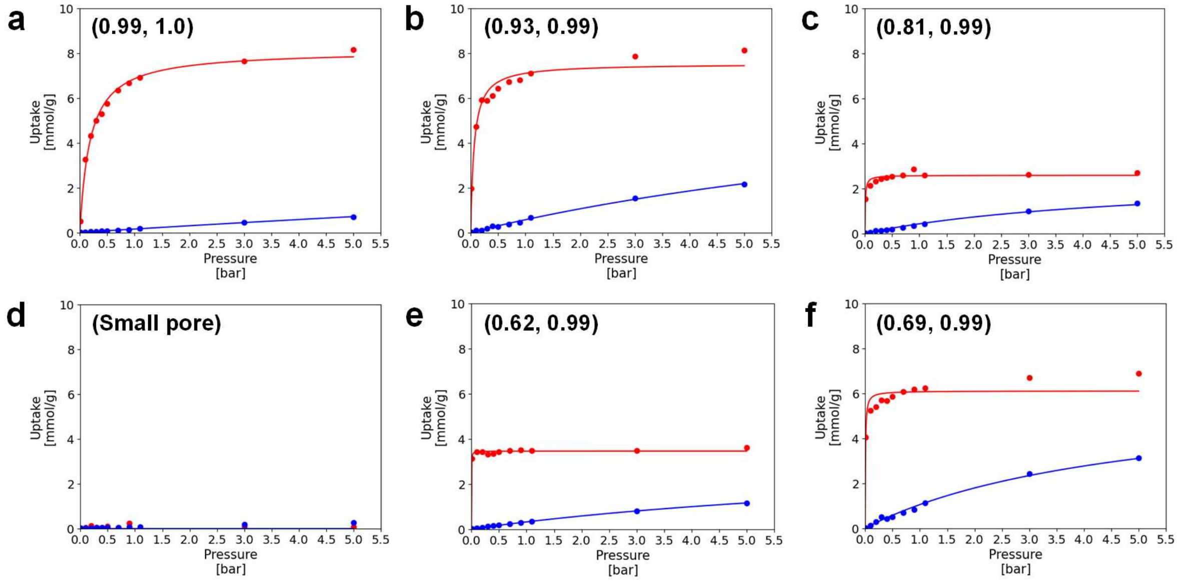

Figure S1. Characteristic examples of isotherms succeeding or failing according to the defined fitting procedure: **(a-c)** successful fitting examples with R^2 higher than 0.80, and **(d-f)** failed fitting examples with one or more R^2 values less than 0.80.

Of the 23,599 porous MOFs studied (> 2.0 Å), 19,158 MOFs were successfully fit to SSL models (R^2 > 0.80) while 4,419 MOFs failed (R^2 < 0.80). Typical examples for successful and failed fitting are shown in **Figure S1 (a-c) and (d-f)**, respectively. One might be curious about why that many MOFs failed in fitting. Further investigations revealed that most failed in fitting because they possess small pores with no uptake or almost no uptake with noisy isotherm (**Figure S1d**). This result is related to our previous screening step to tell if a MOF is porous or not. As mentioned in the method section, a conservative criterion (2.0 Å) for pore limiting diameter (PLD) was adopted to avoid losing MOFs such as CALF-20 and NbOFFIVE-1-Ni which were previously reported in the literature as promising CO_2 capture materials. Their PLDs calculated by the same tool used in this work were 2.75 Å and 2.65 Å, respectively. However, if a commonly used criterion (3.3 Å, a kinetic diameter of CO_2) was used, 9,151 MOFs with PLD less than 3.3 Å would have been discarded in the porous MOF screening.

Instead, 5,557 MOFs (61%) of these 9,151 small-pore MOFs were successfully fit to SSL isotherm and the remaining 3,594 MOFs failed because of too low uptake and noisy isotherm. Therefore, we effectively retrieved those ~5,500 successful MOFs by employing a conservative PLD criterion. Out of 4,419 MOFs which failed in the fitting step from our current workflow, 3,594 MOFs failed because of the small pores with no uptake or almost no uptake with very noisy results.

The remainder MOFs (~800 MOFs) with larger pore than 3.3Å generally failed in fitting because of either sharp and flat CO_2 isotherm (**Figure S1e**) or isotherms which require more complicated isotherm model (**Figure S1f**) to be accurately described. For the former case, the

CO_2 uptake of those MOFs is already saturated at the lowest pressure in the GCMC calculations (0.01bar); thus, their CO_2 isotherm appears very flat. In this case, the R^2 value (coefficient of determination) could be lower than our fitting criteria because of the way it is calculated. The equations defining the R^2 are as follows:

$$R^2 = 1 - \frac{SS_{res}}{SS_{tot}}$$

$$SS_{res} = \sum_i (y_i - f_i)^2$$

$$SS_{tot} = \sum_i (y_i - \bar{y}_i)^2$$

Where y_i, f_i and $\bar{y}_i$ means GCMC uptake at a pressure, uptake from isotherm model at the same pressure and the average of GCMC data over the pressures, respectively. If CO_2 isotherm is flat like **Figure S1e**, the SS_{tot} value could be very small near 0 leading to low R^2 values. In this case, we can use additional GCMC points at lower pressure than the current lowest GCMC pressure (0.01 bar) for fitting. However, those MOFs will require lower pressure than 0.01 bar to desorb CO_2. Haghpanah et al.[20] showed that low evacuation pressure is the main reason for high energy consumption and the pressure lower than 0.02 bar is often difficult to achieve in an industrial column. Also, Figure 3 and 4 in our manuscript show the MOFs with flat isotherms are not suitable for P/VSA process. Therefore, those MOFs will not pass the process performance screening anyway.

The other remaining MOFs (**Figure S1f**), which might fit using a more complicated model isotherm with more parameters, might perform well in P/VSA process (Of course, might not). For these MOFs, their isotherm cannot be fit to current isotherm functional (SSL) under our fitting range corresponding to training set range of our process ML models. As we can see in **Figure S1f,** the fitted isotherm has deviations in both low and high pressure regions, which may lead to inaccuracy in process modeling. In this work, we are limiting our study to only those MOFs where they can be fit in SSL because of a practical reason stated in the method section. Therefore, we are not going to consider those MOFs in this specific work.

S3. Validation of Process Optimization Using ML Models

To validate the role of the MAPLE model in our proposed workflow, the process optimization results for the 15% CO_2 flue gas condition derived from the GA-optimizer using the detailed model and the GA-optimizer using the ML model were compared. For GA-detailed runs, the same GA parameters with GA-MAPLE (e.g., population size of 192, maximum generation of 30, etc.) were used. One of the important things is the GA-detailed code also employs the same grid sampling initialization as the ML models to find the minimum more quickly and accurately. The grid sampling was achieved by the ML model and the 96 best operating conditions were selected as half of the initial populations. The other half was generated from a Latin hypercube sampling in the same way with GA-optimizer using MAPLE ML models. Tests where GA runs were initially seeded with or without this enhanced grid search protocol were performed, ultimately determining that including this grid sampling was beneficial in generating lower optimized values which better correlated with the true minimum values.

100 experimental MOFs were randomly sampled depending on the energy consumption from the process optimization using MAPLE model and the isotherm parameters from the 100 MOFs were used for the process optimization using the GA-detailed code to find a minimum achievable energy consumption. The energy ranges of the samples and the number of samples were tabulated in Table S3. The energy from GA-MAPLE and GA-detailed were compared in Figure S2.

Overall, the optimized energies from ML models and the detailed model have quite linear correlations even though they do not exactly achieve parity (i.e. y=x). Importantly, all the MOFs which have energy lower than 200 kWh/ton from detailed model also have the energy lower than 200kWh/ton from ML model. So, if we want to find the MOFs with energy lower than 200kWh/ton from the detailed model, the MOFs should have the energy lower than 200kWh/ton from ML model at least. Of course, the converse is not true and some MOFs with the energy from ML models less than 200 kWh/ton have slightly higher energy from the detailed model; however, the general trend shows that the surrogate model for the process optimization can be used for the large scale screening thereby significantly reducing the computation time.

Table S3. The GA-MAPLE Energy Ranges of the 100 Samples and the Number of Samples in Each Range

Energy consumption from process optimization using MAPLE model [kWh/tonne]	Number of samples
176.6 *	10
~ 180	10
180 ~ 190	10
190 ~ 200	10
200 ~ 250	10
250 ~ 300	10
300 ~ 350	10
350 ~ 400	10
400 ~	10
NON PRT MOFs	10

* lower bound of the practical limit of energy consumption

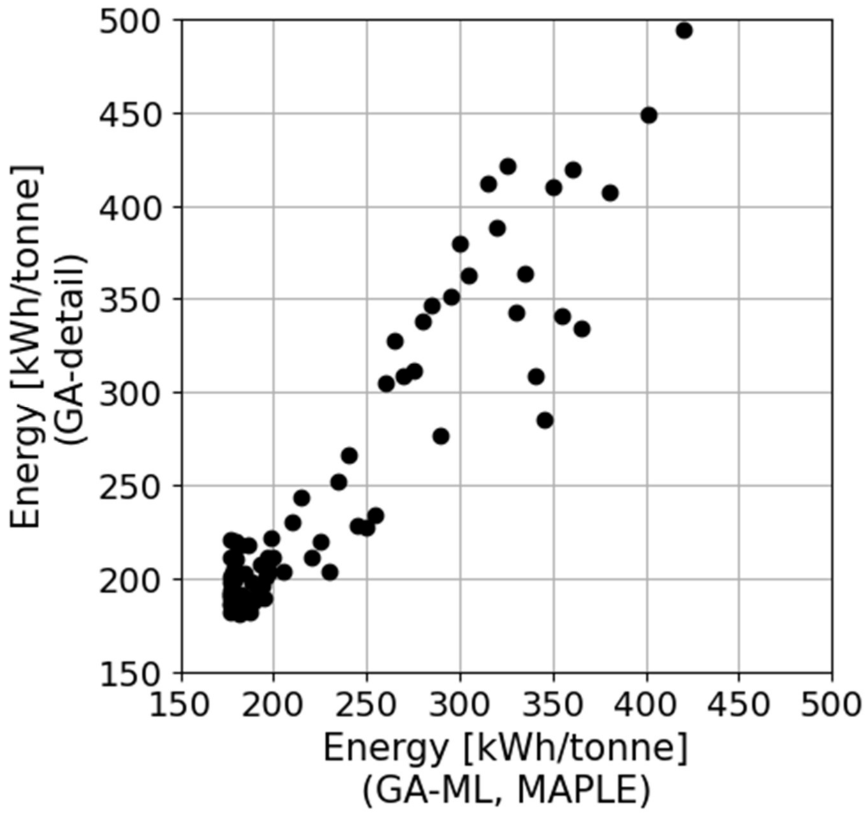

Figure S2. Parity plot of the minimum achievable energy consumption for 100 experimental MOFs obtained from process optimization using ML model (MAPLE) and the detailed model.

S4. Result of Process Optimization Targeting Productivity Maximization at Three Different CO_2 Compositions

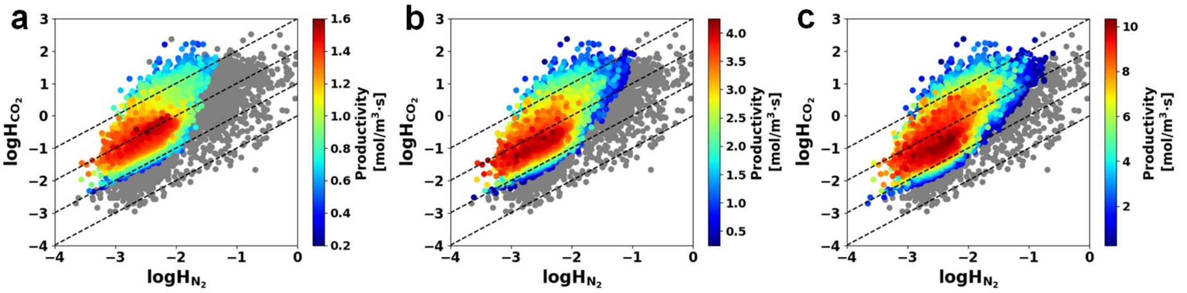

Figure S3. Plot of Henry's law constant of CO_2 and N_2 for 19,158 experimental MOFs with optimized (maximum) productivity in a P/VSA process for three different dry flue gas compositions: **(a)** CO_2 6% and N_2 94%, **(b)** CO_2 15 % and N_2 85% and **(c)** CO_2 35% and N_2 65%. The color mapping represents the maximum productivity achievable while satisfying the purity and recovery targets (PRT, 95% CO_2 purity and 90% recovery). Note productivity scale is different for each CO_2 concentration. The gray points indicate that the MOF cannot meet purity-recovery constraints under any operating conditions. The dashed lines represent lines of constant selectivity calculated as the ratio of the Henry's constants of CO_2 and N_2. Selectivity lines corresponding to values of 1, 10, 100, and 1000, respectively, are depicted from the bottom to the top of each figure.

S5. The Distributions of the Largest Cavity Diameters (LCDs) for Both the Process Top Performers and the Final Top 50 MOFs across Each CO_2 Concentration Case

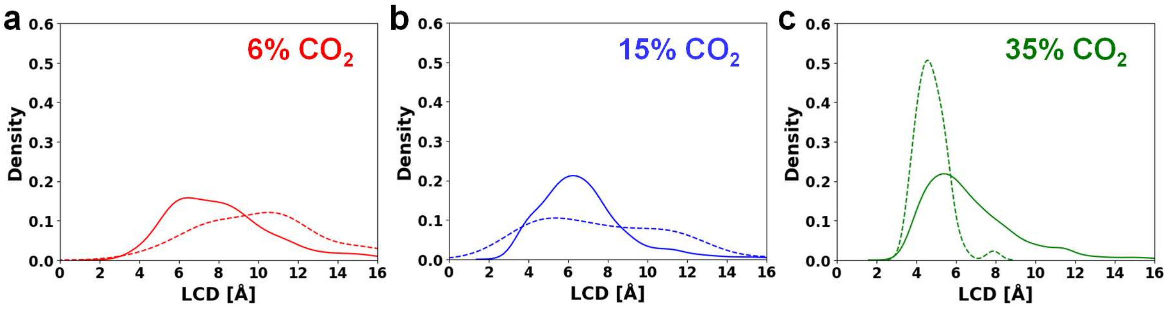

Figure S4. Distribution of largest cavity diameters (LCDs) for MOFs that are within 4% of the PE limit for CO_2 dry flues (solid), compared to the distribution of the top 50 MOFs ranked in terms of the CO_2/H_2O uptake ratio (dashed) at 40%RH that also retain at least 90% of their dry CO_2 uptake capacity. These distributions are shown for **(a)** 6% CO_2 **(b)** 15% CO_2 and **(c)** 35% CO_2 flues.

S6. Visualization of 1D Channel MOFs among the Final Top 50 MOFs at Three Different CO_2 Compositions

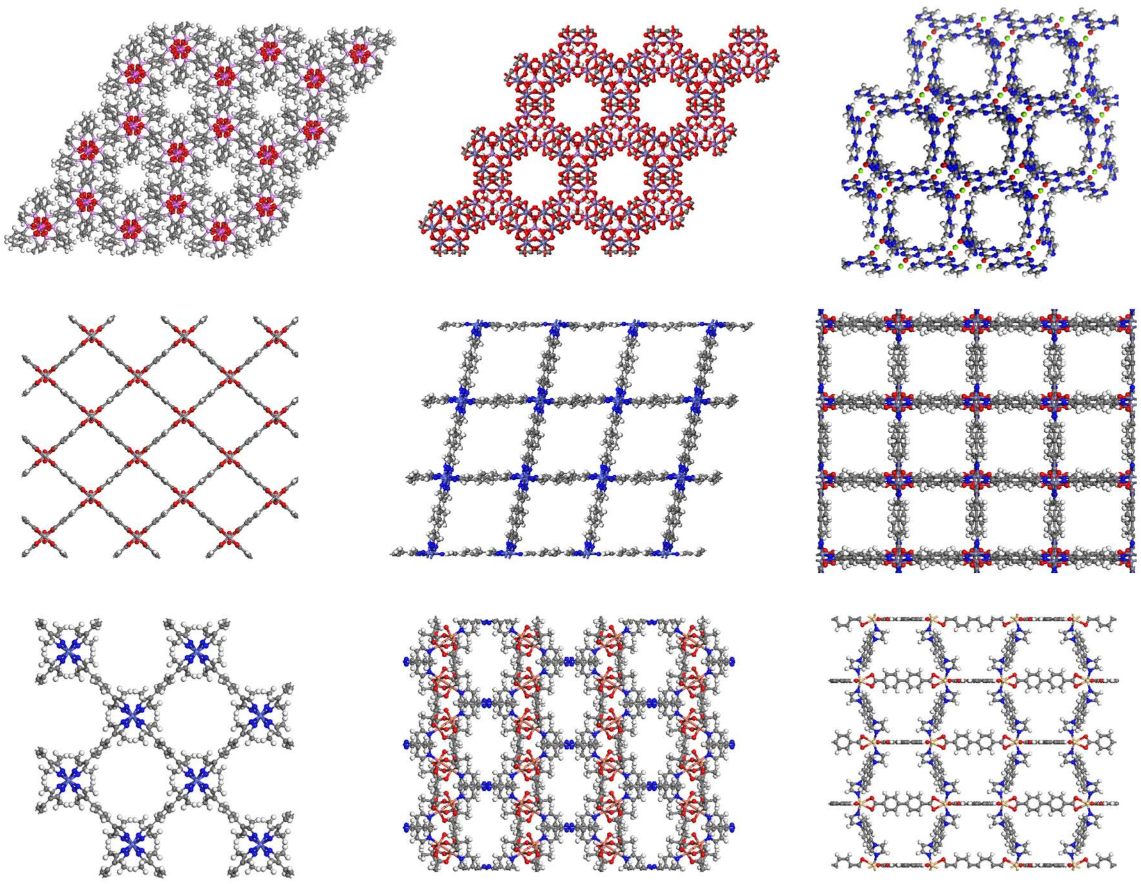

Figure S5. Representative 1D Channel MOFs observed in the 6% CO_2 composition top performer lists.

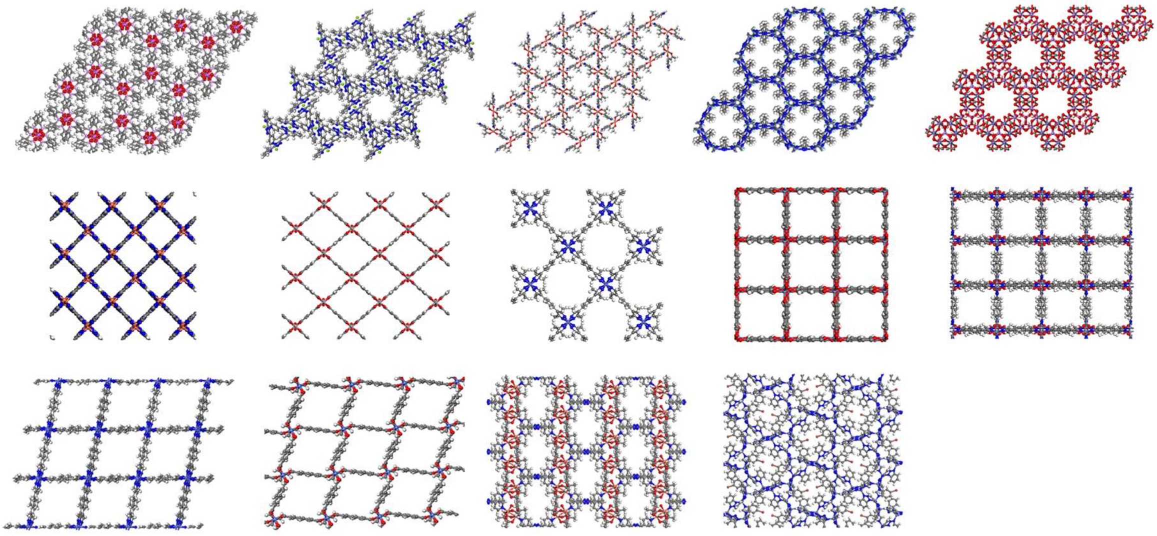

Figure S6. Representative 1D Channel MOFs observed in the 15% CO_2 composition top performer lists.

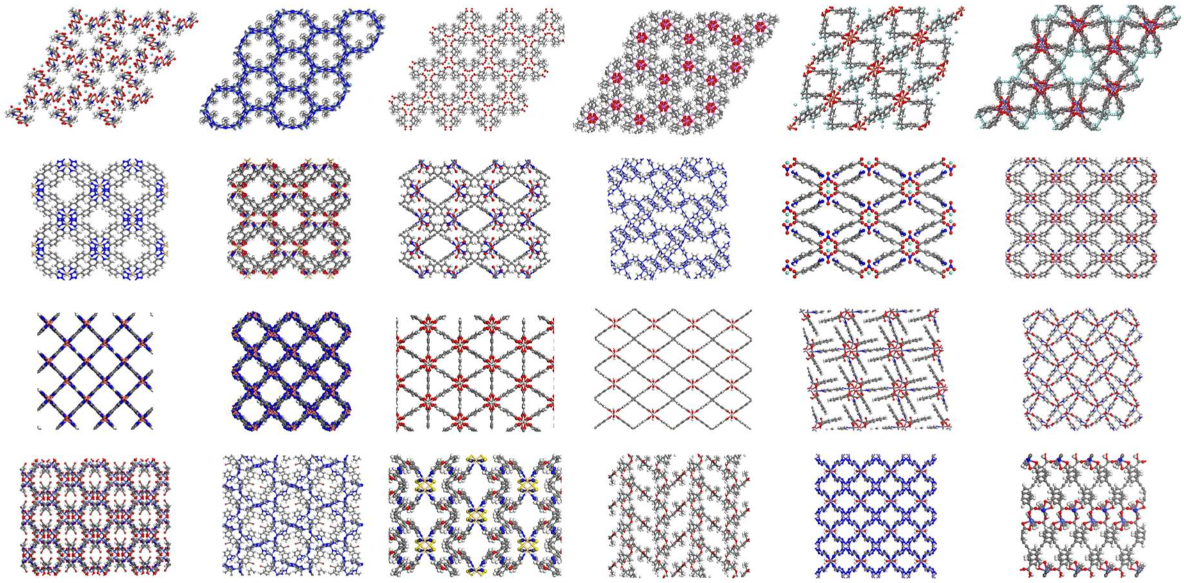

Figure S7. Representative 1D Channel MOFs observed in the 35% CO_2 composition top performer lists.

S7. Process Performances and GCMC Results under Humid Conditions for CALF-20, NbOFFIVE-1-Ni and IISERP-MOF2

From a literature search, SSL parameters were only available for IISERP-MOF-2 among the three considered MOFs. These previously obtained SSL parameters were employed for IISERP-MOF-2 and parameters obtained from GCMC calculations were employed for CALF-20 and NbOFFIVE-1-Ni. Using those SSL parameters, process performances were calculated using the process optimizer combined with MAPLE models and the results are summarized in **Table S4**.

Table S4. Process Performances for CALF-20, NbOFFIVE-1-Ni and IISERP-MOF2

	Purity and Recovery target	Energy (6% case) [kWh/tonne]	Energy (15% case) [kWh/tonne]	Energy (35% case) [kWh/tonne]
CALF-20	✓	665.3	204.5	74.3
NbOFFIVE-1-Ni	–	–	–	–
IISERP-MOF2	✓	525.6	179.5	64.7

CALF-20 exhibits reasonably good performance, although its energy consumption exceeds our defined practical limit. In contrast, NbOFFIVE-1-Ni shows a sharp CO_2 isotherm due to its saturation at very low pressure which leads to very high average internal energy of adsorption values for CO_2 (54 kJ/mol). Unfortunately, this ΔU_{CO_2} is outside the ML model's training set ranges therefore our ML models cannot be used for prediction. However, based on our analysis, this type of MOF where CO_2 uptake is already saturated at the lowest pressure of GCMC is likely to have high energy consumptions in P/VSA process. On the other hand, NbOFFIVE-1-Ni is well-known for its exceptionally high CO_2 uptake at very low pressures, making it a promising candidate for direct air capture combined with temperature swing-based processes. IISERP-MOF2 shows energy consumption within the practical limit across all CO_2 compositions. As previously reported, its extremely low N_2 uptake significantly contributes to the reduced energy demand.

Next, CO_2 retainability under humid flue gas conditions were evaluated (**Table S5**). In conclusion, all three MOFs showed a significant decrease in CO_2 uptake in the presence of water from our simulation results. It is worth noting that we chose a strict condition of 40% relative humidity (RH) as the screening threshold. This corresponds to the pressure value where the binary CO_2 uptake of CALF-20 begins to fall below that of water (40% RH). Therefore, the screening condition used in this study is relatively stringent and enables us to qualitatively assess which MOFs are likely to perform well under humid flue gas conditions.

Table S5. Multicomponent GCMC Results for CALF-20, NbOFFIVE-1-Ni and IISERP-MOF2

CO_2 composition in the flue gas (6%)	CO_2 uptake (dry) [mmol/g]	CO_2 uptake (wet) [mmol/g]	Water uptake (wet) [mmol/g]	CO_2 uptake ratio (wet/dry)	CO_2/water uptake ratio (wet)
CALF-20	1.96	0.11	9.64	0.056	0.011
NbOFFIVE-1-Ni	2.35	0.16	4.48	0.068	0.035
IISERP-MOF2	2.66	0.07	10.03	0.028	0.007
CO_2 composition in the flue gas (15%)	CO_2 uptake (dry) [mmol/g]	CO_2 uptake (wet) [mmol/g]	Water uptake (wet) [mmol/g]	CO_2 uptake ratio (wet/dry)	CO_2/water uptake ratio (wet)
CALF-20	2.56	0.19	9.95	0.075	0.019
NbOFFIVE-1-Ni	2.36	0.35	4.10	0.146	0.084
IISERP-MOF2	3.32	0.16	9.96	0.049	0.016
CO_2 composition in the flue gas (35%)	CO_2 uptake (dry) [mmol/g]	CO_2 uptake (wet) [mmol/g]	Water uptake (wet) [mmol/g]	CO_2 uptake ratio (wet/dry)	CO_2/water uptake ratio (wet)
CALF-20	3.06	1.52	5.39	0.497	0.282
NbOFFIVE-1-Ni	2.36	0.49	3.82	0.206	0.127
IISERP-MOF2	3.76	0.28	9.84	0.075	0.029

References

(1) Gibaldi, M.; Kapeliukha, A.; White, A.; Woo, T. K. Incorporation of Ligand Charge and Metal Oxidation State Considerations into the Computational Solvent Removal and Activation of Experimental Crystal Structures Preceding Molecular Simulation. *J. Chem. Inf. Model.* **2025**, *65* (1), 275–287. https://doi.org/10.1021/acs.jcim.4c01897.

(2) White, A. J.; Gibaldi, M.; Burner, J.; Mayo, R. A.; Woo, T. K. High Structural Error Rates in "Computation-Ready" MOF Databases Discovered by Checking Metal Oxidation States. *J. Am. Chem. Soc.* **2025**, *147* (21), 17579–17583. https://doi.org/10.1021/jacs.5c04914.

(3) Widdowson, D.; Mosca, M. M.; Pulido, A.; Cooper, A. I.; Kurlin, V. Average Minimum Distances of Periodic Point Sets – Foundational Invariants for Mapping Periodic Crystals. *MATCH Commun. Math. Comput. Chem.* **2022**, *87* (3), 529–559. https://doi.org/10.46793/match.87-3.529W.

(4) Widdowson, D. E.; Kurlin, V. A. Resolving the Data Ambiguity for Periodic Crystals. *Adv. Neural Inf. Process. Syst.* **2022**, *35* (NeurIPS), 1–14.

(5) Campañá, C.; Mussard, B.; Woo, T. K. Electrostatic Potential Derived Atomic Charges for Periodic Systems Using a Modified Error Functional. *J. Chem. Theory Comput.* **2009**, *5* (10), 2866–2878. https://doi.org/10.1021/ct9003405.

(6) Burner, J.; Luo, J.; White, A.; Mirmiran, A.; Kwon, O.; Boyd, P. G.; Maley, S.; Gibaldi, M.; Simrod, S.; Ogden, V.; Woo, T. K. ARC-MOF: A Diverse Database of Metal-Organic Frameworks with DFT-Derived Partial Atomic Charges and Descriptors for Machine Learning. *Chem. Mater.* **2023**, *35* (3), 900–916. https://doi.org/10.1021/acs.chemmater.2c02485.

(7) Kresse, G.; Hafner, J. Ab Initio Molecular-Dynamics Simulation of the Liquid-Metal-Amorphous-Semiconductor Transition in Germanium. *Phys. Rev. B* **1994**, *49*, 14251.

(8) Kresse, G.; Joubert, D. From Ultrasoft Pseudopotentials to the Projector Augmented-Wave Method. *Phys. Rev. B* **1999**, *59*, 1758.

(9) Perdew, J. P.; Burke, K.; Ernzerhof, M. Generalized Gradient Approximation Made Simple. *Phys. Rev. Lett.* **1996**, *77* (18), 3865–3868. https://doi.org/10.1103/PhysRevLett.77.3865.

(10) Blöchl, P. E. Projector Augmented-Wave Method. *Phys. Rev. B* **1994**, *50* (24), 17953–17979. https://doi.org/10.1103/PhysRevB.50.17953.

(11) Rappe, A. K.; Casewit, C. J. J.; Colwell, K. S. S.; Goddard, W. A.; Skiff, W. M. UFF, a Full Periodic Table Force Field for Molecular Mechanics and Molecular Dynamics Simulations. *J. Am. Chem. Soc.* **1992**, *114* (25), 10024–10035. https://doi.org/10.1021/ja00051a040.

(12) García-Sánchez, A.; Ania, C. O.; Parra, J. B.; Dubbeldam, D.; Vlugt, T. J. H. H.; Krishna, R.; Calero, S. Transferable Force Field for Carbon Dioxide Adsorption in Zeolites. *J. Phys. Chem. C* **2009**, *113* (20), 8814–8820. https://doi.org/10.1021/jp810871f.

(13) Provost, B. An Improved N2 Model for Predicting Gas Adsorption in MOFs and Using Molecular Simulation to Aid in the Interpretation of SSNMR Spectra of MOFs,

University of Ottawa, 2014.

(14) Horn, H. W.; Swope, W. C.; Pitera, J. W.; Madura, J. D.; Dick, T. J.; Hura, G. L.; Head-Gordon, T. Development of an Improved Four-Site Water Model for Biomolecular Simulations: TIP4P-Ew. *J. Chem. Phys.* **2004**, *120* (20), 9665–9678. https://doi.org/10.1063/1.1683075.

(15) Pai, K. N.; Prasad, V.; Rajendran, A. Practically Achievable Process Performance Limits for Pressure-Vacuum Swing Adsorption-Based Postcombustion CO 2 Capture. *ACS Sustain. Chem. Eng.* **2021**, *9* (10), 3838–3849. https://doi.org/10.1021/acssuschemeng.0c08933.

(16) Pai, K. N.; Prasad, V.; Rajendran, A. Generalized, Adsorbent-Agnostic, Artificial Neural Network Framework for Rapid Simulation, Optimization, and Adsorbent Screening of Adsorption Processes. *Ind. Eng. Chem. Res.* **2020**, *59* (38), 16730–16740. https://doi.org/10.1021/acs.iecr.0c02339.

(17) Haghpanah, R.; Majumder, A.; Nilam, R.; Rajendran, A.; Farooq, S.; Karimi, I. A.; Amanullah, M. Multiobjective Optimization of a Four-Step Adsorption Process for Postcombustion CO2 Capture via Finite Volume Simulation. *Ind. Eng. Chem. Res.* **2013**, *52* (11), 4249–4265. https://doi.org/10.1021/ie302658y.

(18) Moghadam, P. Z.; Li, A.; Liu, X. W.; Bueno-Perez, R.; Wang, S. D.; Wiggin, S. B.; Wood, P. A.; Fairen-Jimenez, D. Targeted Classification of Metal-Organic Frameworks in the Cambridge Structural Database (CSD). *Chem. Sci.* **2020**, *11* (32), 8373–8387. https://doi.org/10.1039/d0sc01297a.

(19) Chung, Y. G.; Haldoupis, E.; Bucior, B. J.; Haranczyk, M.; Lee, S.; Zhang, H.; Vogiatzis, K. D.; Milisavljevic, M.; Ling, S.; Camp, J. S.; Slater, B.; Siepmann, J. I.; Sholl, D. S.; Snurr, R. Q. Advances, Updates, and Analytics for the Computation-Ready, Experimental Metal–Organic Framework Database: CoRE MOF 2019. *J. Chem. Eng. Data* **2019**, *64* (12), 5985–5998. https://doi.org/10.1021/acs.jced.9b00835.

(20) Haghpanah, R.; Nilam, R.; Rajendran, A.; Farooq, S.; Karimi, I. A. Cycle Synthesis and Optimization of a VSA Process for Postcombustion CO_2 Capture. *AIChE J.* **2013**, *59* (12), 4735–4748. https://doi.org/10.1002/aic.14192.
